# Inhibition of Osteoblast Differentiation by JAK2^V617F^ Megakaryocytes Derived From Male Mice With Primary Myelofibrosis

**DOI:** 10.3389/fonc.2022.929498

**Published:** 2022-07-08

**Authors:** Aikaterini Karagianni, Shinobu Matsuura, Louis C. Gerstenfeld, Katya Ravid

**Affiliations:** ^1^ Department of Internal Medicine, University of Crete, School of Medicine, Heraklion, Greece; ^2^ Department of Medicine, Whitaker Cardiovascular Research Institute, Boston University School of Medicine, Boston, MA, United States; ^3^ Department of Orthopaedic Surgery, Boston University School of Medicine, Boston, MA, United States

**Keywords:** primary myelofibrosis, megakaryocyte, bone, osteosclerosis, osteoblast, JAK2, mouse

## Abstract

Past studies described interactions between normal megakaryocytes, the platelet precursors, and bone cell precursors in the bone marrow. This relationship has also been studied in context of various mutations associated with increased number of megakaryocytes. The current study is the first to examine the effects of megakaryocytes from transgenic mice carrying the most common mutation that causes primary myelofibrosis (PMF) in humans (JAK2^V617F^) on bone cell differentiation. Organ level assessments of mice using micro-computed tomography showed decreased bone volume in JAK2^V617F^ males, compared to matching controls. Tissue level histology revealed increased deposition of osteoid (bone matrix prior mineralization) in these mutated mice, suggesting an effect on osteoblast differentiation. Mechanistic studies using a megakaryocyte-osteoblast co-culture system, showed that both wild type or JAK2^V617F^ megakaryocytes derived from male mice inhibited osteoblast differentiation, but JAK2^V617F^ cells exerted a more significant inhibitory effect. A mouse mRNA osteogenesis array showed increased expression of Noggin, Chordin, Alpha-2-HS-glycoprotein, Collagen type IV alpha 1 and Collagen type XIV alpha 1 (mostly known to inhibit bone differentiation), and decreased expression of alkaline phosphatase, Vascular cell adhesion molecule 1, Sclerostin, Distal-less homeobox 5 and Collagen type III alpha 1 (associated with osteogenesis) in JAK2^V617F^ megakaryocytes, compared to controls. This suggested that the mutation re-programs megakaryocytes to express a cluster of genes, which together could orchestrate greater suppression of osteogenesis in male mice. These findings provide mechanistic insight into the effect of JAK2^V617F^ mutation on bone, encouraging future examination of patients with this or other PMF-inducing mutations.

## 1 Introduction

Myeloproliferative Neoplasms (MPNs) are a heterogeneous group of chronic hematological diseases that arise from the clonal expansion of abnormal hematopoietic stem cells. Depending on the affected clones, these cells produce excess numbers of red blood cells, white blood cells, and/or platelets, leading to a hypercellular bone marrow (BM). Primary myelofibrosis (PMF), the MPN with the worst prognosis, has an annual incidence of 0.47 per 100,000 and a median survival of 4.4 years (1, 2). It is accompanied by an increase in abnormal megakaryocytes (MKs), myelofibrosis, and splenomegaly due to extramedullary hematopoiesis (3). Although, osteosclerosis -the excess formation of bone within the BM– has been considered a serious complication, the effect of PMF on bone health is not entirely clear, given that few reports did not observe increased bone volume in PMF patients compared to controls (reviewed in (4)). Different methods of bone assessment, difficulty of recruiting the right controls, and different lifestyle/medication of patients that may affect bone could account for possible discrepancies in results reported in different studies (4).

In order for bone to develop, mesenchymal stromal cells (MSCs) must first commit to the osteoblastic lineage by differentiating into immature pre-osteoblasts (pre-OBs) and subsequently into mature osteoblasts (OBs). These cells are responsible for the synthesis and secretion of most proteins of the bone extracellular matrix (ECM). The initial unmineralized organic portion of the bone matrix is called osteoid, which then becomes mineralized (5). The whole procedure is orchestrated by transcription factors and a number of genes that are considered as markers of osteogenesis. Runt-related transcription factor 2 (RUNX2), the master regulator of OB differentiation, is the earliest transcription factor necessary for committing mesenchymal progenitors to the osteoblastic lineage (pre-OBs). Osterix (OSX) acts downstream of RUNX2 and is required for the final commitment of progenitors to pre-osteoblasts and the activation of osteoblast-specific genes. Type 1 collagen (COL1) and alkaline phosphatase (ALP) become highly expressed during subsequent stages of OB differentiation. Finally, when pre-OBs become fully differentiated OBs, there is elevated expression of bone sialoprotein (BSP), osteocalcin (OC) and osteopontin (OPN) (5).

Bone consists of a dense outer shell of cortical bone which forms a shealth around the inner marrow space that is composed of thin spicules of trabecular bone which has extensive contact with the hematopoietic elements and vascular network of the marrow. MKs only occasionally are seen associated with the endosteum surface or cortical bone and show an atypical localization near the paratrabecular areas and endosteal border in myelodysplastic and myeloproliferative conditions (4, 6–8). Kacena et al. were the first to report that mice deficient in GATA-1 and NF-E2 transcription factors, leading to an increased number of abnormal, immature MKs, have a striking increase in bone volume (9). So far, eight mouse models with different gene perturbations and increased number of MKs (TPO^high^; GATA-1^low^; NF-E2^-/-^; Mpl^f/f^/PF4cre; Lnk^-/-^; Mpig6b^-/-^; Mpig6b^f/f/^Gp1ba-Cr^+/KI^; Pt-vWD) were shown to develop osteosclerosis in their long bones. However, the gender of the mice utilized to assess the bone phenotype was not specified (9–12), or only females were used for analyses (13–15), or females presented with a worse bone phenotype compared to males (16, 17). In other studies, results were combined as a whole for the two sexes (18, 19). All these mouse models were kept on a C57BL/6 background, with the exception of NF-E2 deficient mice that were on a 129/Sv background, and GATA-1^low^ mice (10) that were on a mixed C57BL/6-129Sv/CD1 background. Yet, a number of studies reported that normal MKs inhibit OB differentiation in co-culture systems (20–23), whereas other groups reported the opposite (24, 25). Possible explanations to these discrepancies might be differences in species, sex and age of animals used, and source of cells.

The effect of the most common human mutation that causes PMF (JAK2^V617F^) on MKs' ability to affect bone differentiation has not been studied. To investigate this property, we used a PMF mouse model, the Vav1-hJAK2^V617F^ (denoted as JAK2^V617F^ mice) transgenic mice that expresses the major driver-mutation of PMF in humans in hematopoietic cells, and recapitulate the most phenotypes seen in patients, including megakaryocytosis (26). In the current study we sought to investigate the direct effect of JAK2^V617F^ mutated MKs on osteoblast differentiation, finding that male MKs have an inhibitory effect on early OB programming. In accordance, male JAK2^V617F^ mice have reduced bone volume.

## 2 Materials and Methods

### 2.1 Animals

JAK2^V617F^ mice (26), constitutively expressing the Vav1-hJAK2V617F transgene (generous gift of Dr. Zhizhuang Joe Zhao from University of Oklahoma), were backcrossed with C57BL/6J (Jackson laboratory), brought to homozygosity and used post 10 generations of breeding. Age-matched C57BL/6J wild-type mice served as controls. Male mice were utilized for all experiments. All protocols were approved by the Boston University Medical Campus Institutional Animal Care and Use Committee (IACUC).

### 2.2 Micro-Computed Tomography

The right femur of 30-week old male JAK2^V617F^ or WT mice was dissected with the femoral head intact, wrapped in in phosphate buffered saline (PBS)-soaked gauze and stored at -20°C until micro-computed tomography (micro-CT) was conducted. A high-resolution desktop micro-tomographic imaging system (μCT40, Scanco Medical AG, Brüttisellen, Switzerland) was used to assess trabecular bone architecture in the distal femoral metaphysis and cortical bone morphology of the femoral mid-diaphysis. Scans were acquired using a 10 μm^3^ isotropic voxel size, 70 kVP, 114 μA, 200 ms integration time, and were subjected to Gaussian filtration and segmentation. Image acquisition and analysis protocols adhered to the Journal of Bone and Mineral Research (JBMR) guidelines for the assessment of rodent bones using micro-CT. In the femur, trabecular bone microarchitecture was evaluated in a 1.5 mm (150 transverse slices) long region of the distal metaphysis beginning approximately 200 μm superior to the peak of the growth plate and extending proximally. The trabecular bone regions were identified by manually contouring the endocortical region of the bone. A threshold of 400 mgHA/cm^3^ was used to segment bone from soft tissue. Trabecular microarchitecture was analyzed using the standard trabecular bone morphology script in the Scanco Evaluation Program. Cortical bone was assessed in 50 transverse micro-CT slices (500 μm long region) at the femoral mid-diaphysis and the region included the entire outer most edge of the cortex. Cortical bone was segmented using a fixed threshold of 700 mgHA/cm^3^. Blood cell profiles and spleen weights of the mice used for analysis are shown in **Supplementary Tables 1** **,** **2**, depicting a typical profile of PMF (3).

### 2.3 Three-Point Bending

The same femurs used for micro-CT, were mechanically tested in three-point bending using an electrical force material testing machine (Electroforce 3230, Bose Corporation, Eden Prairie, MN). The bending fixture had a bottom span length of 8 mm. The test was performed with the load point in displacement control moving at a rate of 0.1 mm/sec with force and displacement data collected at 60 Hz. All of the bones were positioned in the same orientation during testing with the cranial surface resting on the supports and being loaded in tension. Bending rigidity (EI, Nmm^2^), apparent modulus of elasticity (Eapp, GPa), ultimate moment (Mult, Nmm), apparent ultimate stress (σapp, MPa) work to fracture (Wfrac, mJ), and apparent toughness (Uapp, mJ/mm^3^) were calculated based on the force and displacement data from the tests and the mid-shaft geometry measured with micro-CT. Work to fracture is the energy that was required to cause the femur to fracture, and it was calculated by finding the area under the force-displacement curve using the Riemann Sum method. Bending rigidity was calculated using the linear portion of the force-displacement curve. The minimum moment of inertia (Imin) was used when calculating the apparent material properties.

### 2.4 Bone Tissue Histology- RGB Staining

The left femur of the mice used for micro-CT and three-point bending was fixed with 4% PFA for two days at 4°C, and then decalcified with 20% EDTA at 4°C for two weeks. EDTA solution was changed three times per week. Bones were then paraffin processed and 5 μm sections were cut. Slides were deparaffinized (3 times Xylene 3 min/each), dehydrated (100% EtOH 5 min, 100% EtOH 2 min, 95% EtOH 2 min, 75% EtOH 2 min) and transferred to dH_2_O for 5 min. RGB staining (RGB being the acronym for the three primary color used: picrosirius *R*ed, fast *G*reen and alcian *B*lue) was performed as in (27): 20 min 1% Alcian Blue 8GX (Sigma, #A-3157) in 3% aqueous acetic acid solution pH 2.5 (American Bioanalytical, # 64-19-7), 5 min wash with dH_2_O, 20 min 0.04% Fast Green FCF in dH_2_O (Fischerscientific, #BP123-10), 5 min wash with dH_2_O, 30 min picrosirius red (Polysciences, #24901B-250), 5 min wash with two changes of 1% acetic acid in dH_2_O. Sections were then dehydrated (2 times 4 dips in 95% EtOH, 4 dips in 100% EtOH, 5 min in 100% EtOH), cleared in Xylene (2 times, 2 min each) and mounted in permount mounting medium (Fischerscientific, #SP15-100). Images were acquired with whole slide scanner Olympus VS120.

### 2.5 Complete Blood Count, Bone Marrow Histology and Reticulin Staining

Blood from the retro-orbital plexus of mice used for micro-CT,three-point bending, and histology was collected with EDTA-coated capillaries and tubes, and complete blood counts were obtained using HEMAVET (Drew Scientific). Bone marrow tissue sections were prepared and stained with hematoxylin & eosin, and when indicated, modified Gomori’s silver staining was carried out to assess reticulin deposition. These procedures were performed as we described in an earlier study (28).

### *2.6 In-Vitro* Differentiation of MKs From BM

Total BM was flushed from femurs and tibia of mice using Minimum Essential Medium alpha (MEMα) (Gibco, #12571063) with 10% fetal bovine serum (FBS) (Sigma, #F0926), 50 units/ml penicillin, and 50 μg/ml streptomycin (Gibco, #15140-122). Cells were filtered through 100 μm cell strainer and cultured (37°C, 5% CO_2_) as 10^7^ cells/ml in 6-well plates (Falcon Corning, #353046), in MEMα, with 10% FBS, 50 units/ml penicillin, 50 μg/ml streptomycin and 25 ng/ml pegylated megakaryocyte growth and development factor (PEG-MGDF, gift of Kirin Brewery, Japan). MKs were purified after 4 days of culture, using a two-step bovine serum albumin (BSA) gradient, as described in (29), followed by cell counting.

### 2.7 *In-Vitro* Differentiation of OB Progenitors From BM and OB-MK Co-Cultures or OB-MK Conditioned Medium Cultures

BM cells were flushed as previously described. Cells were filtered through 70 μm and then 40 μm cell strainers and cultured (37°C, 5% CO_2_) as 13.3x10^6^ cells per well of 6-well plate, in MEMα with 10% FBS, 50 units/ml penicillin, and 50 μg/ml streptomycin. After 4 days of culture, half of the media was replaced with fresh media, and on day 6 the whole media was replaced with media containing the following osteo-induction mix: 10 nM dexamethasone (Sigma, #D4902), 50 μM L-ascorbic acid (Sigma, #A7506) and 8 mM B-glycerophosphate (Santa Cruz, #SC-203323) as described in previous studies (30, 31). This later culture is referred to as an OB culture. In parallel cultures, mouse BM derived from JAK2^V617F^ or age-matched control mice were differentiated to MKs, as described above. On day 8 of the OB culture, the whole media was replaced with fresh media containing induction mix, and 2x10^4^ MKs (JAK2^V617F^ or control wild type (WT)) were added in each well containing pre-OBs. After three days of co-culture, MKs were removed and adhering cells were washed three times with warm phosphate buffered saline (PBS) to remove residual MKs. The cells were then scraped in lysis buffer supplied with the RNeasy Plus Micro kit (Qiagen, #74034), and RNA isolation was performed according to manufacturer’s protocol.

In experiments involving OB-MK conditioned medium (CM), mouse BM was cultured under MK promoting conditions (as above), and MKs were purified on day 3 (using a BSA gradient as above), and reseeded as 2x10^4^ MKs in 3ml of media consisted of MEMα, 10% FBS, 50 units/ml penicillin, 50 μg/ml streptomycin and 25 ng/ml PEG-MGDF. After one day, the MKs were removed by centrifugation at 1000 rpm, for 5 min and MK-CM was added to the OB culture, also as described above.

### 2.8 Quantitative RT-PCR

#### 2.8.1 OB-MK Co-Culture Experiments

500 ng RNA was used for complementary DNA (cDNA) preparation with Quantitect Reverse transcription cDNA kit (Qiagen, #205311). Taqman probes used to access mouse OB differentiation were as follows: Runx2 (Mm00501580_m1), Osterix/Sp7 (Mm00504574_m1), Collagen1a1 (Mm00801666_g1), and Alkaline phosphatase liver/bone/kidney (Mm00475834_m1).

#### 2.8.2 Testing Various mRNAs in MKs

1 μg RNA was used for cDNA preparation (Qiagen, #205311). Taqman probes used: Noggin (Mm01297833_s1), Vcam1 (Mm01320970_m1), Chordin (Mm00438203_m1), Bmp2 (Mm01340178_m1), and Alkaline phosphatase liver/bone/kidney (Mm00475834_m1).

The obtained CT values were normalized to those obtained for the housekeeping 18S ribosomal RNA (rRNA) (Hs99999901_s1), using the ΔΔCT method, and the relative expression was determined by the 2^-ΔΔCT^ equation. All probes were predesigned TaqMan assays, purchased from Applied Biosystems.

#### 2.8.3 Screening of MKs for Bone Related mRNAs

The commercially available Mouse Osteogenesis RT² Profiler PCR Array (Qiagen, #PAMM-026Z) was used for comparing the expression of JAK2^V617F^ vs. control MKs for 84 genes related to the development of the skeletal system as well as bone mineral metabolism. MKs were prepared as previously described. 1 μg RNA was used for cDNA preparation with RT² First Strand Kit (Qiagen, #330401). RT² SYBR Green qPCR Mastermix (Qiagen, #330500) was mixed with cDNA based on manufacturer’s protocol and loaded. Analysis was conducted with an automated template provided by the manufacturer (Qiagen), and all data were normalized to the average of 5 housekeeping genes: β-actin, β-2 microglobulin, glyceraldehyde-3-phosphate dehydrogenase, β-glucuronidase and heat shock protein 90-alpha.

All RT-PCR experiments were conducted on an Applied Biosystems Viia7 life technologies machine.

### 2.9 Western Blotting

Total BM cells from JAK2^V617F^ and control mice were differentiated to MKs as previously described. On day 3, MKs were purified with a two-step BSA gradient and 7x10^4^ MKs were reseeded in 500 μl media without FBS in 48-well non-tissue culture treated plates (Falcon, #351178) for 24h. MK culture supernatant was concentrated to a final volume of 30 μl using microcon-10 kDa centrifugal filter unit with Ultracel-10 membrane (Millipore, #MRCPRT010) and MKs from each well were lysed with lysis buffer (50 mM Tris HCI pH 7.6, 150 mM NaCl, 1% Triton-X, 0.5% sodium deoxycholate and 1 mM EDTA pH 7.4). The supernatants, the whole volumes of protein samples (that were lysed in same volume of lysis buffer) as well as 0.5 ng recombinant mouse Noggin protein (abcam, # 200277) were subjected to SDS−PAGE on 12% polyacrylamide gel and transferred to polyvinylidene fluoride membrane (PVDF). The membrane was blocked for 1.5 hours at room temperature (RT) with a 5% (w/v) non−fat milk solution in TBS−0.1% Tween−20 (20 mM Tris and 140 mM NaCI, pH 7.6). Subsequently, it was subjected to overnight incubation at 4°C, rocking with primary antibodies against Noggin (used at 2 ug/ml, Abcam, #239520) or β-actin (used at 1:10000 dilution, Sigma, #A5441), followed by incubation for 1.5 hours at RT with horseradish peroxidase (HRP) conjugated anti−mouse secondary antibody (1:1000 for Noggin and 1:4000 for β-actin, Cell-Signaling, #7076S). All primary and secondary antibody solutions were made in 0.5% (w/v) non−fat milk in TBS−0.1% Tween−20. The membrane was incubated with Immobilon Western Chemiluminescent HRP Substrate (Millipore, # WBKLS0100) in a 1:1 mixture for 1 min and bands were visualized using the chemiluminescence detection system ImageQuant LAS4000 (GE Healthcare).

### 2.10 Flow Cytometry Analysis

BM from femurs and tibia of mice was flushed with cold buffer containing 0.5% BSA and 2 mM EDTA in calcium and magnesium-free PBS. Freshly isolated BM cells were strained with 100 μm cell strainer, and 2x10^6^ cells were used for analysis. For analysis of cell surface expression of Vcam1 in MKs, cells were stained for 15 min with anti-CD106 FITC (eBioscience, #11-1061-81), anti-CD41 PE (eBioscience, #12-0411-83), and Zombie Aqua (Biolegend, #423102), for live/dead discrimination. All antibodies were used at 1:200 dilution, except anti-CD106 which was used at 1:100. Data was acquired on BD LSRII (BD Biosciences) and analyzed on FlowJo, version 10.8.1 from BD Biosciences.

### 2.11 Statistical Analysis

Statistical analyses were performed by unpaired two-tailed t-test with Excel or Graphpad Prism software (Version 6.01 for Windows, Graphpad Software Inc., CA, USA). All data are shown as mean± standard deviation. P-values<0.05 were considered statistically significant.

## 3 Results

### 3.1 JAK2^V617F^ Male Mice Have Decreased Bone Volume

**Figure 1:**
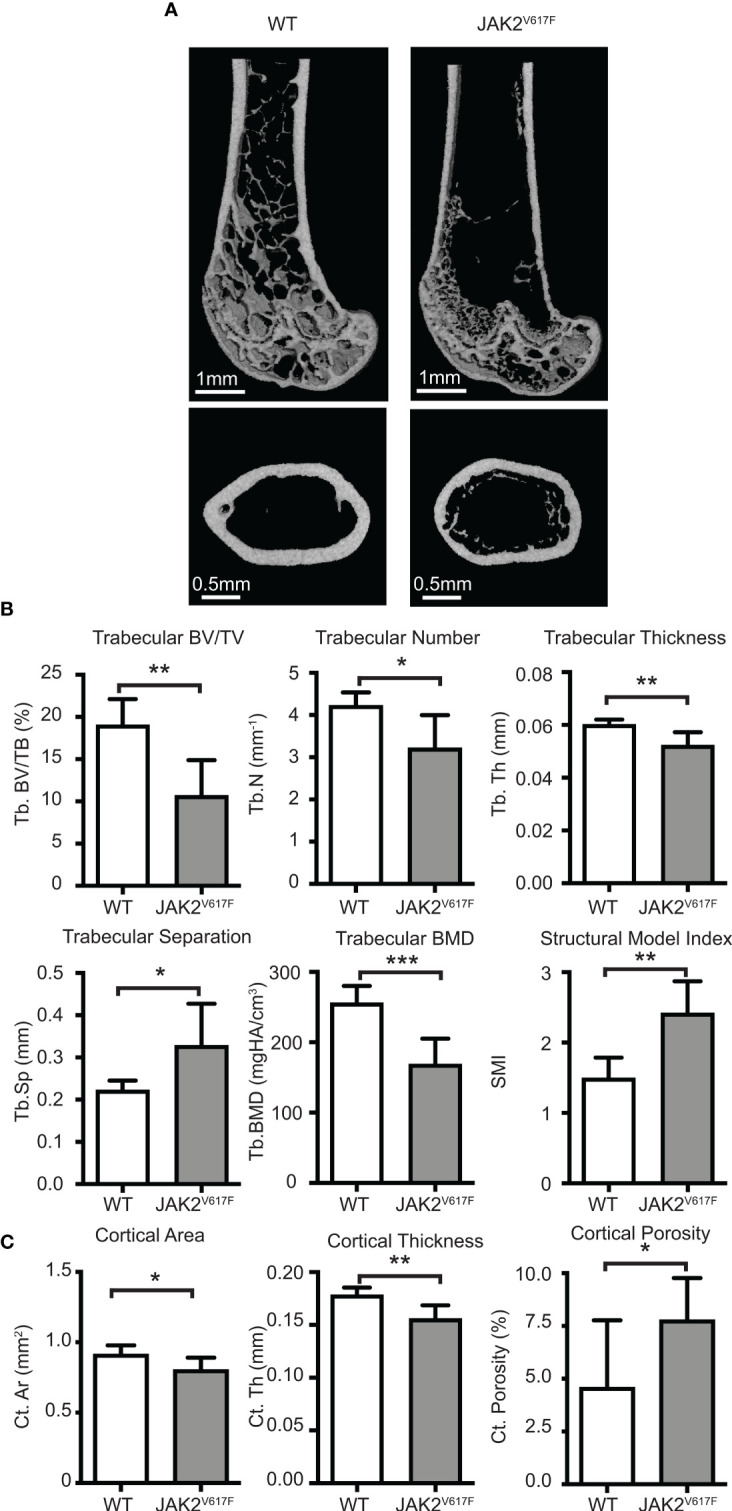
Micro-CT analyses. Femurs of JAK2^V617F^ mice and matching controls were subjected to micro-CT analysis. **(A)** Representative micro-CT images. The upper panel is showing the distal femoral metaphysis and the lower panel is showing the femoral mid-diaphysis. **(B)** Quantitative micro-CT analysis of trabecular structure. Measurements include trabecular bone volume fraction (Tb.BV/TV, %), trabecular bone mineral density (Tb. BMD, mgHA/cm^3^), trabecular thickness (Tb.Th, mm), trabecular number (Tb.N, mm^-1^), trabecular separation (Tb.Sp,mm), and structural model index (SMI). **(C)** Quantitative micro-CT analysis of cortical structure. Measurements include cortical bone area (Ct.Ar, mm^2^), cortical thickness (Ct.Th, mm), and cortical porosity (Ct. Porosity, %). The rest of the cortical measurements can be found in **Supplementary Figure 2**. Measurements are presented as mean ± SD. Seven JAK2^V617F^ and six WT 30 weeks old male mice were analyzed, at an age where potential effect on bone might be more fully manifested. *Denotes p<0.05; **Denotes p<0.01; ***Denotes p<0.001.

In our earlier publications we showed that the JAK2^V617F^ mouse model used in the current study develops several hallmarks of PMF, including a fibrotic marrow noted already at 15 to 16 weeks of age (32, 33), in accordance with the original publication of this mouse phenotypes (26). Here, we confirmed these properties, as evident by reticulin and hematoxylin & eosin staining of bone marrow sections, cellularity, increased spleen size and blood cell count (**Supplementary Figure 1** **, and** **Supplementary Tables 1** **and** **2**). This PMF mouse model was used to examine the effect of the JAK2^V617F^ mutation on bone volume. Preliminary micro-CT studies showed a trend towards bone gain in female mice (not shown), but a significant reduction in bone volume in the mutated male mice (**Figure 1**). Since increased bone volume was studied and reported before for MK-driven myelofibrosis in female mice (such as in mice deficient in GATA-1 or NF-E2), and since our data with male mice presented a unique example of bone loss due to this mutation that was not reported before, we chose to focus on understanding the male phenotype.

Micro-CT analysis of bones showed that the JAK2^V617F^ mutation is associated with a significant decrease in trabecular bone in males compared to matching controls (**Figures 1A, B**). Trabecular bone volume fraction (Tb.BV/TV), bone mineral density (Tb.BMD), number (Tb.N) and thickness (Tb.Th) were all decreased in JAK2^V617F^ male mice compared to age-matched controls. At the femur mid-diaphysis, the JAK2^V617F^ mice had lower cortical area (Ct.Ar) and thickness (Ct.Th), and higher cortical porosity (Ct. Porosity) (**Figures 1A, C**). There was also a trend towards having lower moments of inertias (**Supplementary Figure 2**), which is one of the main indicators of bending strength. For this reason, we proceeded to mechanical testing (three-point bending) of the same femurs that were used for micro-CT, to determine if there were differences in whole bone mechanical properties. JAK2^V617F^ femurs had slightly lower bending rigidity than WT, but no significant differences were observed between the groups (**Supplementary Figure 3**). This finding is in agreement with the micro-CT results for the femoral diaphysis for the male mice, which showed that male JAK2^V617F^ mice had slightly lower cortical area and cortical thickness and a trend towards lower minimum moment of inertia (this is the moment of inertia about the axis in which the femurs were bent during the testing). It is possible that bone mechanical properties might be different in the two experimental groups under conditions of extreme demand for tissue regeneration, such as bone fracture.

### 3.2 Trabecular Bone of JAK2^V617F^ Male Mice is Hypomineralized

**Figure 2:**
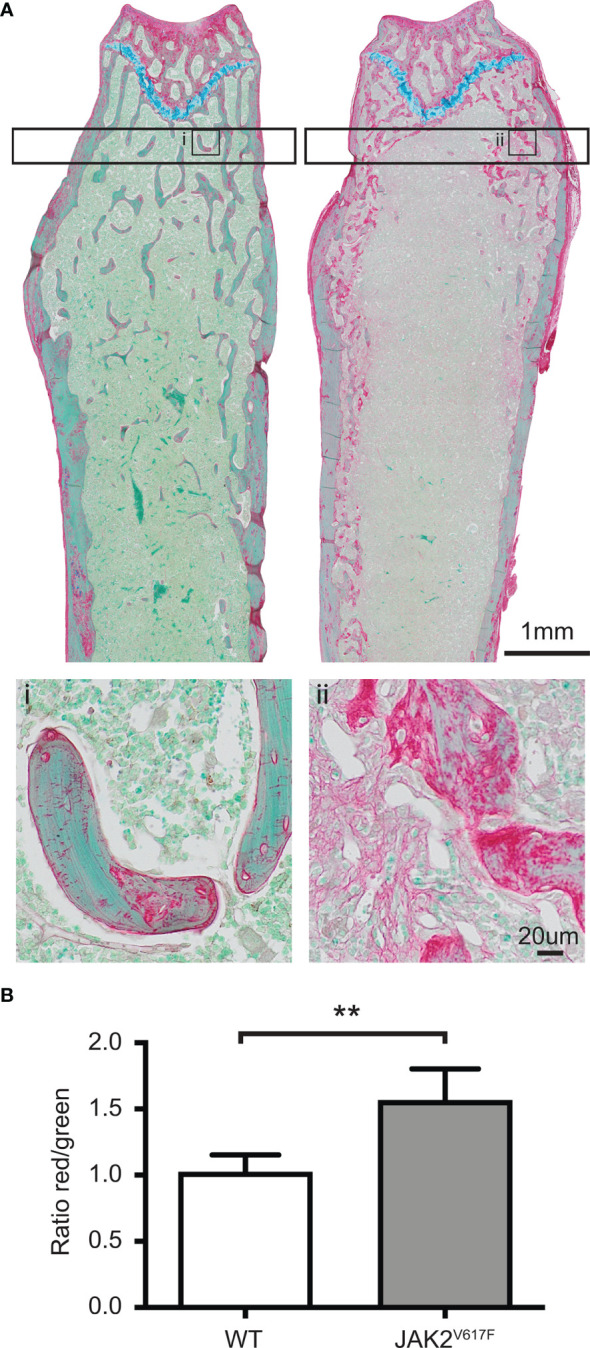
RGB trichrome staining of femurs. Femurs of JAK2^V617F^ male mice and matching controls were stained with picrosirius Red, fast Green and alcian Blue, providing discrimination between mineralized and unmineralized (osteoid) bone. **(A)** Representative images of bone sections of WT mice (denoted as i) and JAK2^V617F^ (denoted as ii). Osteoid appears red, mineralized bone appears green and cartilage appears blue. The lower part of Panel A shows a magnification of an inset in the upper part of the panel. **(B)** Quantitative analysis of the ratio osteoid (red)/mineralized bone (green) for trabecular bone. Measurements are presented as mean ± SD. The analysis of the trabecular bone was conducted 50 μm distant from the edge of the growth plate, extending 300 μm. Four JAK2^V617F^ and four WT 30 weeks old male mice were analyzed. **Denotes p<0.01.

We next assessed the bones at tissue level. This analysis showed that in tissues derived from JAK2^V617F^ males there was more osteoid, which is the unmineralized organic portion of the bone matrix that forms prior to the maturation of bone tissue, compared to WT. Indeed, the ratio of osteoid (red) to mineralized bone (green) for trabecular bone was increased 53% in the JAK2^V617F^ males compared to age-matched controls (**Figures 2A, B**).

### 3.3 JAK2^V617F^ MKs Derived From Male Mice Exert an Inhibitory Effect on OB Differentiation

Based on the observations of increased osteoid and less bone, we envisioned a possible effect of the mutated MKs on OB differentiation. To assess this contention, co-culture studies were carried out. WT BM cells were cultured and induced to differentiate to OBs, using an induction cocktail in presence or absence of MKs for three days, followed by measurement of markers of OB differentiation. Longer incubation of MKs under these conditions was avoided, as we found that within 5 days of the co-culture the MKs’ shape and viability appeared compromised. We focused on differentiation markers that are known to peak during the first days of OB programming. It is important to note that the location from which the stromal cells are harvested affects their responsiveness to the MKs (21). Several other studies used murine calvaria cells as source of OB progenitors and murine fetal liver as source of MKs (9, 20, 23, 34). In our study, the source of both OBs and MKs was BM progenitors based on the following reasons: 1) MK-driven osteosclerosis in mouse models is mainly observed in long bones, which means that the mesenchymal progenitors residing in the BM are more likely to differentiate into OBs, 2) the increased number of MKs and MK-progenitors in BM are more likely to interact with the mesenchymal progenitors that are present in this anatomical area, and 3) cells derived from calvaria are already committed to the osteoblast lineage.

**Figure 3:**
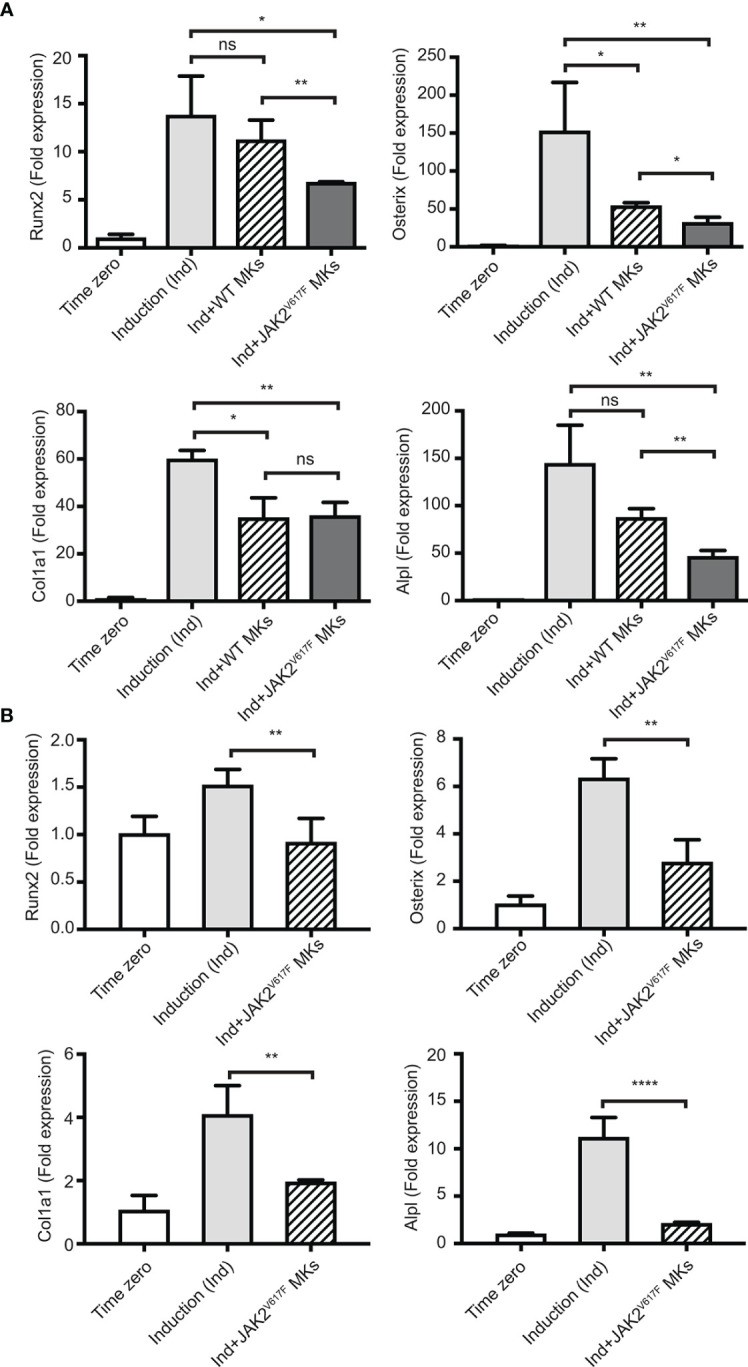
Inhibition of the OB differentiation program by JAK2^V617F^ MKs. Bone marrow cells were cultured and differentiated towards OBs with an induction mix in presence or absence of JAK2^V617F^ mutated or control WT MKs for three days, as described under methods. RNA was isolated from differentiating OBs and quantitative RT-PCR was carried out to detect the expression of early differentiation markers of OBs, including Runx2, Osterix, Col1a1 and Alpl. Data were normalized to 18S rRNA and values were compared to time zero, where this point refers to samples that were isolated right before the beginning of induction of OB differentiation. P-values were acquired with an unpaired two-tailed t-test. **(A)** JAK2^V617F^ MKs (derived from ten 18 weeks old male mice per experiment) inhibit OB differentiation of mesenchymal progenitors (derived from ten 13 weeks old WT mice). Shown is an experiment performed in triplicates, representative of three biological repeats. **(B)** JAK2^V617F^ MKs (derived from four 13 weeks old male mice) inhibit OB differentiation of mesenchymal progenitors derived from JAK2^V617F^ mice (four 14 weeks old male mice per experiment). *Denotes p<0.05; **Denotes p<0.01; ****Denotes p<0.0001.

As shown in **Figure 3A**, the presence of JAK2^V617F^ MKs decreased the expression of all four tested markers of OB differentiation, reaching statistical significance. Runx2, Osterix, Col1a1 and Alpl expression decreased by 2.02-, 4.76-, 1.66-, and 3.10- fold, respectively. The effect of WT MKs on OB differentiation was less consistent between the experiments, as also observed in previous studies (35). In our system, WT MKs had a tendency to decrease the expression of OB differentiation markers, however, usually without reaching statistical significance. Runx2, Osterix, Col1a1 and Alpl markers showed a 1.15-, 2.81-, 1.70-, and 1.65- fold decrease, respectively, but only Osterix and Col1a1 had a p-value<0.05. In accordance with data shown in **Figure 3A**, JAK2^V617F^ MKs derived from male mice inhibited OB differentiation when the BM cells were derived from male JAK2^V617F^ mice. Indeed, JAK2^V617F^ MKs decreased the expression of Runx2, Osterix, Col1a1 and Alpl markers by 1.65-, 2.26-, 2.09-, and 5.28- fold, respectively (**Figure 3B**). Thus, MKs derived from male JAK2^V617F^ mice exert an inhibitory effect on OB programming.

**Figure 4:**
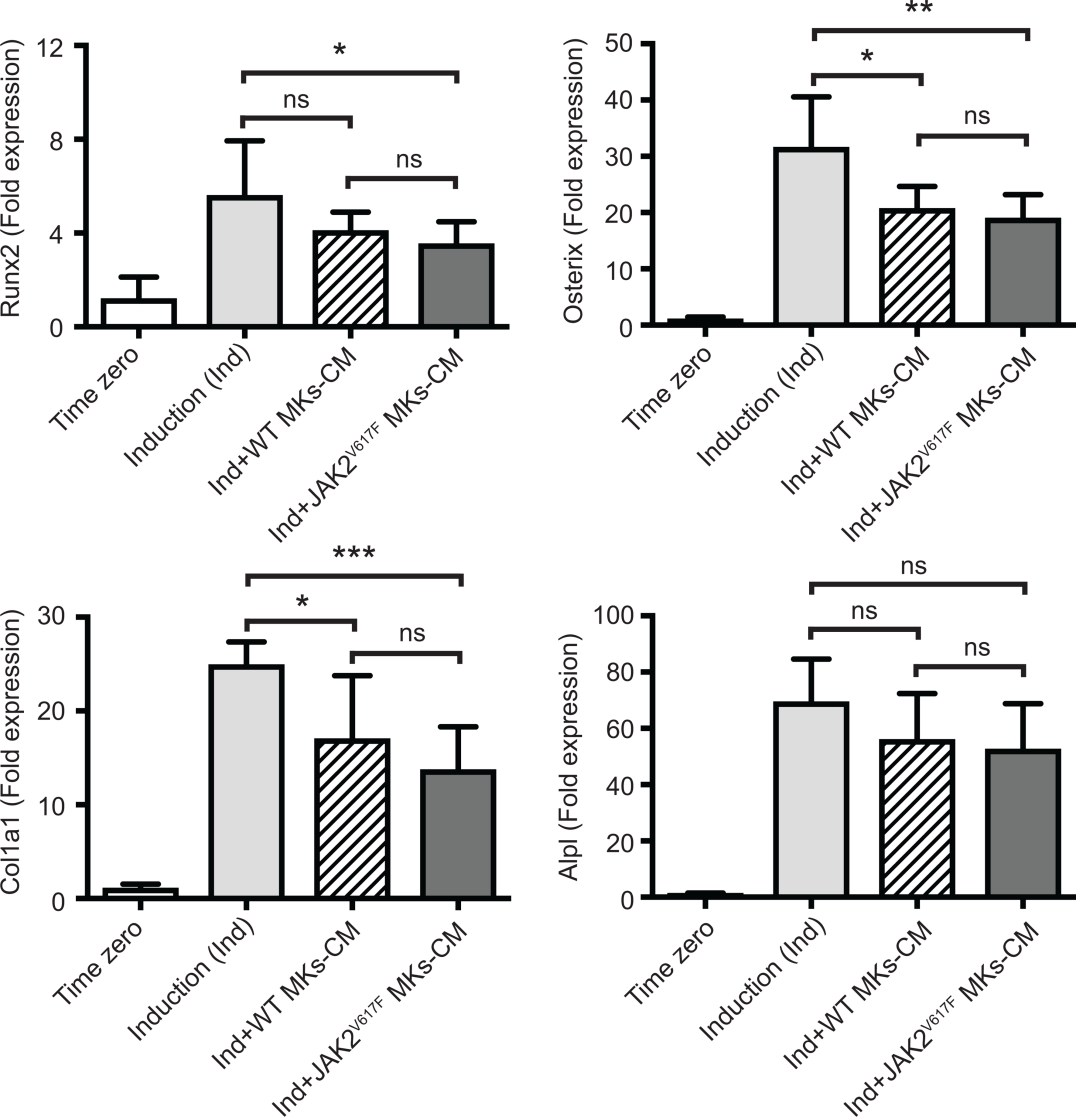
Effect of MK-conditioned medium (CM) on the OB differentiation program. Bone marrow cells were cultured and differentiated towards OBs with an induction mix in presence or absence of MK culture supernatants derived from JAK2^V617F^ mutated or control WT cultures, as described under methods. RNA was isolated from differentiating OBs and quantitative RT-PCR was carried out to detect expression of OB differentiation markers, Runx2, Osterix, Col1a1 and Alpl. Data were normalized to 18S rRNA and values were compared to time zero, where this point refers to samples that were isolated right before the beginning of induction of OB differentiation. Results shown are averages of 2 biological repeats, each done in triplicates, and for each, several mice were pooled. For the OB culture, a total of twenty five 13 weeks old control male mice were used, and MKs were derived from a total of twenty three or twenty six control or JAK2^V617F^ male mice, respectively, each 18 weeks old. P-values were generated with an unpaired two-tailed t-test. *Denotes p<0.05; **Denotes p<0.01; ***Denotes p<0.001.

We next sought to address whether the MK inhibitory effect was mediated by direct cell to cell contact or by secreted factors. MK-derived conditioned medium (MK-CM), prepared as described under methods, was used instead of MKs in the co-cultures. As shown in **Figure 4**, WT MK-CM tended to decrease Runx2, Osterix, Col1a1 and Alpl markers by 1.37-, 1.53-, 1.46-, and 1.24- fold, respectively, with only Osterix and Col1a1 having a p-value<0.05. On the other hand, the inhibitory effect of the CM from JAK2^V617F^ MKs was statistically significant for all markers, except Alpl, and the decrease corresponded to 1.58-, 1.66-, 1.82-, 1.32- fold for Runx2, Osterix, Col1a1 and Alpl. This suggested that JAK2^V617F^ MKs exert their effect on OB differentiation, likely owing to secreted factors, although cell contact could contribute as well.

### 3.4 mRNA Expression Screening of JAK2^V617F^ MKs Supports an OB-Inhibitory Profile

**Figure 5:**
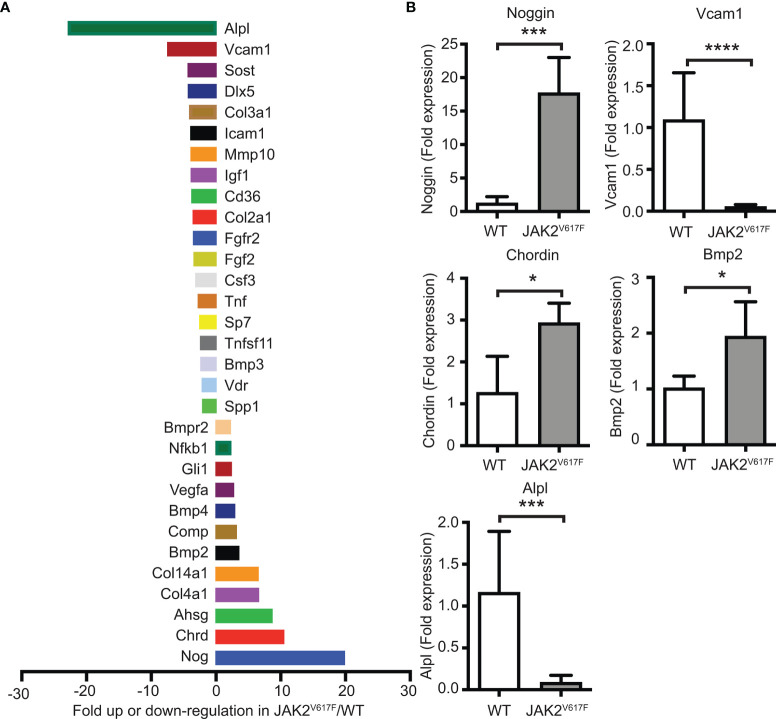
Differential expression of bone development-related genes in MKs of JAK2^V617F^ mice compared to WT mice. **(A)** Graphical representation of more than 2-fold difference expression between JAK2^V617F^ and WT MKs, using the commercial RT² Profiler™ PCR Array Mouse Osteogenesis PAMM-026Z. Bone marrow cells were differentiated towards MKs as described under methods. After 4 days of culture, MKs were purified with a two-step BSA gradient and RNA-isolation and RT-PCR were performed using 1 ug of RNA. Results were normalized to β-actin, β-2 microglobulin, glyceraldehyde-3-phosphate dehydrogenase, β-glucuronidase and heat shock protein 90-alpha. Four 17 weeks old control male mice and four 17 weeks old JAK2^V617F^ male mice were analyzed. MKs for 2 mice were combined as one sample to obtain sufficient material and increase biological representation, and, thus, 2 samples were tested per category. The whole gene panel and full names of mRNA encoded genes can be found in **Supplementary Table 3**. **(B)** Confirmation of mRNA results obtained with the commercial mouse osteogenesis array. Relative expression of highly displayed mRNAs in the experimental groups, including Noggin, Vcam1, Chordin, Bmp2 and Alpl in JAK2^V617F^ MKs, was confirmed using quantitative RT-PCR analysis with Taqman primers and 1 ug of RNA. Data were normalized to 18S rRNA. Four 21 weeks old JAK2^V617F^ mice and five control male mice were individually cultured, representing one sample each. *Denotes p<0.05; ***Denotes p<0.001; ****Denotes p<0.0001.

In an attempt to understand which genes could be possible candidates responsible for the greater inhibition of OB differentiation seen in the cultures by JAK2^V617F^ MKs, a commercially available mRNA mouse osteogenesis array was selected to test the expression of 84 different genes functioning in the development of the skeletal system and bone mineral metabolism. The array detects transcripts encoding for growth factors and proteins mediating bone cells proliferation and differentiation processes, as well as extracellular matrix molecules and cell adhesion molecules involved in bone development. Differentially expressed transcripts in JAK2^V617F^ MKs vs. matching controls with more than a 2-fold difference are shown in **Figure 5A**, whereas those with more than a 4-fold difference of expression are listed in **Table 1**. The whole array panel is listed in **Supplementary Table 3**. Notably upregulated mRNAs in JAK2^V617F^ MKs compared to control consist of: Alpha-2-HS-glycoprotein (Ahsg) 8.66-fold; Chordin (Chrd) 10.47-fold; Collagen type XIV alpha 1 (Col14a1) 6.50-fold; Collagen type IV alpha 1 (Col4a1) 6.58-fold; and Noggin (Nog) 19.85-fold change. More than 4-fold downregulated mRNA levels in JAK2^V617F^ MKs compared to control MKs consist of: Alkaline phosphatase liver/bone/kidney (Alpl) 22.81-fold; Collagen type III alpha 1 (Col3a1) 4.06-fold; Distal-less homeobox 5 (Dlx5) 4.25-fold; Sclerostin (Sost) 4.30-fold; and Vascular cell adhesion molecule 1 (Vcam1) 7.45-fold. Next, we tested representative mRNAs with Taqman technology to validate the results of the gene expression array, which was based on SYBR Green technology. There was a 13.88-, 2.31-, and 1.91- fold increased expression of Noggin, Chordin and Bmp2, respectively, and a 12.93- and 19.89-fold decreased expression of Alpl and Vcam1 mRNA in JAK2^V617F^ MKs compared to controls (**Figure 5B**). As referenced in **Table 1** and further outlined in the discussion, the cluster of displayed mRNAs point to altered programming of JAK2^V617F^ MKs towards an inhibitory effect on osteogenesis, in accordance with the co-culture data.

**Table 1:** Fold up (“+”)- or down (“-”)-regulation of bone-regulating mRNA transcripts in JAK2^V617F^ MKs vs. WT MKs. mRNAs with a JAK2^V617F^/WT ratio ≥4 are included. (Sec) denotes a secreted protein.

mRNA	Fold up- or down-regulation in JAK2^V617F^/WT	Inhibitory (I) or Stimulatory (S) effect on Bone development (reference)
**Ahsg (Alpha-2-HS-glycoprotein)**	+8.66 (Sec)	I (36–39)
**Alpl (Alkaline phosphatase liver/bone/kidney)**	-22.81	S (40, 41)
**Chrd (Chordin)**	+10.47 (Sec)	I (42, 43)
**Col3a1 (Collagen, type III, alpha 1)**	-4.06 (Sec)	S (44, 45)
**Col4a1 (Collagen, type IV, alpha 1)**	+6.58 (Sec)	possibly I (46–49)
**Col14a1 (Collagen, type XIV, alpha 1)**	+6.50 (Sec)	not known
**Dlx5 (Distal-less homeobox 5)**	-4.25	S (50, 51)
**Nog (Noggin)**	+19.85 (Sec)	I (52, 53)
**Sost (Sclerostin)**	-4.30 (Sec)	I (54)
**Vcam1 (Vascular cell adhesion molecule 1)**	-7.45	possibly S (55–57)

### 3.5 Protein Expression of a Secreted or Cell Surface Protein in JAK2^V617F^ MKs

**Figure 6:**
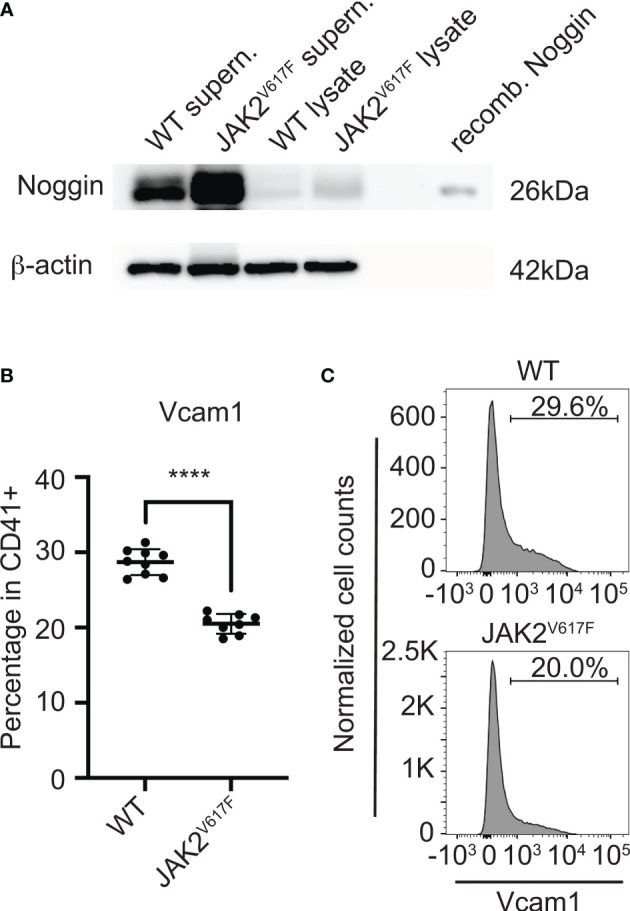
Noggin and Vcam1 proteins are differentially expressed in JAK2^V617F^ MKs compared to control. **(A)** Western blotting confirmed that the level of cellular and secreted Noggin protein is increased in/by the mutated MKs. Bone marrow cells from JAK2^V617F^ and WT mice were differentiated towards MKs and purified as described under methods. MKs were re-plated for another 24h, using the same number of MKs for JAK2^V617F^ and WT samples. Supernatants were concentrated and subjected to SDS-PAGE together with MK lysates, using also recombinant Noggin protein marker as control. Membranes were probed with anti-Noggin or with anti-β-actin (as loading control). Supern. denotes supernatant and recomb. denotes recombinant. Data are representative of five JAK2^V617F^ or five matching control 18 weeks old male mice. **(B)** Flow cytometry analysis showing that Vcam1 protein is significantly decreased in MKs (CD41+) of JAK2^V617F^ mice compared to controls. Eight JAK2^V617F^ male mice and nine control 14 weeks old mice were used. **(C)** Representative data of flow cytometric analysis of Vcam1 expression in MKs shown in **(B)**. ****Denotes p<0.0001.

Considering the effect of MKs or their CM on OB differentiation, and the magnitude of differential mRNA expression of Noggin (a secreted protein) or Vcam1 (a cell surface protein) in JAK2^V617F^ MKs compared to controls, we further tested their expression at protein level. Noggin is a secreted glycoprotein, known to tightly bind a variety of bone morphogenetic proteins (BMPs) and prevent them from binding to their receptors, thus, acting as a BMP-antagonist (58). BMP signaling is highly important for osteogenesis (59). In accordance with the mRNA results, Western blot analysis using MK-derived supernatant (conditioned medium) and MK-cell lysate confirmed that the level of secreted Noggin is higher in JAK2^V617F^ MKs compared to matching controls (**Figure 6A**). As to Vcam1, this protein on the cell surface is important for the adhesion of cells to α4β1 integrin on other cells, as also reported in (55). Immuno-flow cytometry analysis of CD41+ cells (CD41 being a MK marker) showed downregulation of this protein in JAK2^V617F^ MKs compared to controls (**Figures 6B, C**). In addition, the expression of integrin subunits α4 and β1 was confirmed in our OB system by flow cytometry (data not shown). Although RT-PCR showed significant downregulation of Alpl mRNA in MKs from JAK2^V617F^ mice compared to controls, using western blot analysis, we observed no difference in Alpl protein level between JAK2^V617F^ and WT MK cell lysates (data not shown).

## 4 Discussion

A growing body of literature has investigated possible effects of WT MKs on OB differentiation and mechanisms involved (reviewed in (35)). Our current study describes for the first time a reducing effect of JAK2^V617F^ mutation on bone volume in male mice, and the influence of JAK2^V617F^ mutated MKs on early markers of OB differentiation, and possible roles of direct contact vs. secreted factors on this effect. In line with an earlier report of bone gain in female mice with MK-driven myelofibrosis (15), we too observed in preliminary studies a trend for bone gain in 30-week old JAK2^V617F^ female mice. Understanding this exact phenotype in mutated females will include exploring the role of estrogen and/or androgen in affecting the level of change in bone volume, followed by mechanistic analysis to begin to sort out the nature of a potentially different female phenotype. Considering this, and since reduced bone volume in JAK2^V617F^ males was not reported before, our study has focused on exploring mechanisms related to this finding.

So far, only Oikonomidou et al. examined bone phenotypes in JAK2^V617F^ mice, however their mouse model differs from ours (60). The authors used a knock-in mouse model generated by Mullally et al. (61) that expresses the murine JAK2^V617F^ mutation, but recapitulates mostly polycythemia vera instead of PMF. Due to decreased survival rate, the authors transplanted BM from JAK2^V617F^ mice into male irradiated WT mice (the gender of BM donors was not specified) and observed loss of trabecular, but not of cortical bone. They also reported a significant decrease in OB numbers and bone formation, but no differences regarding osteoclast (OC) number and function (60). Importantly, a report that JAK inhibitors increased bone formation in both inflammatory and non-inflammatory bone loss mouse models, as well as under normal steady conditions, could implicate that JAK signaling exerts a negative effect on bone (62). The positive effect of JAK inhibitors on bone repair was also confirmed in two rheumatoid arthritis patients that were treated with JAK inhibitors (62).

In context of mechanisms by which MKs affect bone development, previous studies showed that co-culturing of OB progenitors with WT MKs reduced OB differentiation capacity (20, 21, 23). A MK-induced decrease in OB differentiation was seen when utilizing three types of stromal cells that were generated from two species: rabbit BM, rabbit dental pulp and an immortalized line of mouse bone marrow stromal cells (BMSC) (21). Whether the effect was driven by direct cell contact and/or secreted factors needed to be addressed. The use of 30% (v/v) MK CM derived from the human K562 cell line inhibited the differentiation of a murine osteoblastic cell line MC3T3-E1, showing a possible important contribution of MK-secreted factors, however, without pointing to any possible candidates (22). In contrast, Tang et al. reported enhanced OB-differentiation with both direct contact co-culture and MK-CM, when using murine BM MKs and murine calvarial OBs (24). The authors proposed that secreted TGF-β1 by MKs is an important factor, since the use of a neutralizing antibody and the use of CM from MKs with deleted TGF-β1 weakened the MK-induced stimulatory effect. A positive effect of MKs on OB differentiation was also reported by Bord et al. who observed increased COL1A1 (indication of enhanced OB differentiation), as well as increased OPG and decreased RANKL expression levels, when culturing MKs from human cord blood and OBs from human normal donors’ bone samples for a period of one or two days (25). Nevertheless, they did not observe any significant differences with the use of cell-impermeable inserts, suggesting that direct cell contact is required in order to elicit effects. It should be noted that in all the aforementioned studies the ratio of MKs : OBs for co-cultures was ~1:1, which does not reflect the real BM conditions, given the rarity of BM MKs; Only Bord et al. used a ratio of 1 MK:10 OB (25). Moreover, the sex of species used in these experiments was not specified.

In search for possible candidates responsible for the effect of male-derived JAK2^V617F^ mutated MKs on osteogenesis, we used a mouse mRNA osteogenesis array. Several transcripts were identified as differentially expressed between the two groups, of which major displayed ones were also validated by quantitative RT-PCR. TGF-β, a known promoter of bone development, was reported to mediate MK effect on OB differentiation in culture (24). In our analysis we found no statistically significant differences in the levels of TGF-β mRNA between JAK2^V617F^ and WT MKs. In fact, three isoforms were downregulated in JAK2^V617F^ MKs as follows: TGF-β1 was downregulated by 1.29-, TGF-β2 by 1.61-, and TGF-β3 by 1.07-fold, compared to WT MKs. On the other hand, several transcripts significantly inhibited in JAK2^V617F^ MKs derived from male mice are known to target BMP regulatory activity, with expected effects on bone differentiation.

Alpha 2-Heremans-Schmid glycoprotein (AHSG), also known as fetuin-A (Fet-A) plays a direct role as a physiological inhibitor of bone calcification (36) by inhibiting apatite formation during the mineralization process (37). It also plays an indirect role in inhibiting osteogenesis by its ability to bind and antagonize the function of TGF-β and BMPs (38). The increased osteogenesis observed in Ahsg deficient mice, further supports its inhibitory function (39). Therefore, Ahsg upregulation in JAK2^V617F^ MKs could contribute to stronger OB inhibition. The upregulated expression of Chordin and Noggin also could affect osteogenesis since they are well characterized BMP-antagonists. Chordin inhibits OB differentiation and mineralization (42), and an enhanced osteogenic differentiation and bone regeneration were observed, *in vitro* and *in vivo*, when Chordin was knocked-down (43). Noggin also inhibits OB differentiation, and its overexpression in mice resulted in significantly reduced bone formation and osteoporosis (52, 53). Sclerostin was initially described as a BMP-antagonist as well, however, its ability to antagonize BMPs has been questioned and it is believed that it acts mainly by inhibiting the canonical Wingless and Int-1 (WNT) pathway (54). One would expect to see an upregulation, instead of the observed downregulation of Sclerostin in JAK2^V617F^ MKs. However, the fact that Sclerostin can bind Noggin with high affinity and form a complex that limits the activity of each other as a BMP-antagonist could be relevant to the OB inhibitory effect of JAK2^V617F^ mutated MKs. Based on the osteogenesis array, Noggin expression was upregulated ~20 fold in the JAK2^V617F^ MKs, and a speculation could be that in order for Noggin to fully exert an inhibitory effect on OB differentiation, Sclerostin was downregulated. In summary it is interesting to note that the multiple number of the observed changes in JAK2^V617F^ MKs would directly target BMP regulatory activity, since in previous studies of marrow stromal cells osteogenesis was shown to be driven by the autologous upregulation of BMP expression, that could be blocked by Noggin, addition of blocking antibodies to BMP2 (31) and the knowdown of BMP2 expression (63).

Our analysis also revealed downregulation of transcripts encoding for promoters of osteogenesis. Tissue-nonspecific isozyme of Alpl, the product of *ALPL* gene, plays a key role in the calcification of bones, by hydrolyzing inorganic pyrophosphate to produce inorganic phosphate during the mineralization process (40). Knock-out mice of this gene have serious skeletal defects (41). We noted a significant downregulation in ALP mRNA level in JAK2^V617F^ MKs. Vcam1, a transmembrane glycoprotein mostly found in activated endothelium, is a ligand for α4β1 and α4β7 integrins and is significant for cell-cell interactions (55). Integrin α4β1, which among other cells is also expressed by MSCs, has a potential role in upregulating osteoblastogenesis by enhancing OB differentiation (56, 57). Of note, administration of α4β1 peptidomimetic resulted in an increased bone formation, but the mechanism remains unknown (56). A decreased expression of Vcam1 in JAK2^V617F^ MKs would likely result in decreased signaling through α4β1 integrin and thus decreased OB differentiation. Dlx5 is a transcription factor that stimulates the expression of several OB markers, and was described as master regulator of osteogenesis (50). In addition, Dlx5-deficient mice had serious skeletal abnormalities (51). However, it is not clear how the decreased expression of Dlx5 in JAK2^V617F^ MKs might affect OB differentiation.

Collagen constitutes about 90% of the bone matrix and has an important role by acting as a scaffold for bone cells, regulating cell behavior and modulating the availability of growth factors and cytokines (44). Col3^-/-^ mice had a dramatic decrease in trabecular bone mass, and their mesenchymal progenitors showed reduced capacity to differentiate to OBs *in vitro* (44). In addition, patients with Ehlers-Danlos Syndrome (EDS), an inherited disorder caused by mutations in the *COL3* gene, have reduced bone density (45). Therefore, the downregulation of Col3a1 observed in JAK2^V617F^ MKs could contribute to the decreased OB differentiation in our cultures. COL4A1, a major component of basement membranes that influences their interaction with cells, was found to be upregulated in osteoporotic human bone compared to normal bone, and downregulated during early differentiation of MC3T3-E1 cells in culture (46, 47). The precise role of COL4A1 in OB differentiation is not clear, but it has been suggested that a decrease in COL4A1 during OB differentiation may result in increased free osteogenin, which enhances OB differentiation, thus, acting as an inhibitor of matrix mineralization. Further, COL4A1 can strongly bind TGFβ and osteogenin, which is important for OB differentiation (48, 49). In our experiment, JAK2^V617F^ MKs had increased Col4a1 expression, which could contribute to the inhibitory effect on OB differentiation. In view of our findings, we propose that, together, an array of direct cell-contact and secreted factors that are dysregulated in JAK2^V617F^ MKs contribute to the inhibitory effect on OB differentiation.

Given the large fold-difference in Noggin, Vcam1, and Alpl mRNA levels in JAK2^V617F^ MKs compared to WT MKs, and considering their roles in osteogenesis, we examined their altered expression on protein level. Indeed, there is a significant increase in Noggin and decrease in Vcam1 proteins in JAK2^V617F^ MKs, mirroring the mRNA results. However, there was no difference in Alpl protein level between JAK2^V617F^ and WT MKs, using an anti-Alpl antibody that detects the non-tissue specific form of the protein. There are several tissue isoforms of Alp. Past studies suggested that the Alpl content of MKs may be an index of their physiological activity and an immunoenzymatic staining method, known as anti-alkaline phosphatase (APAAP), became a breakthough for immunophenotyping and diagnosing haematological malignancies (64–67). It is possible that although Alpl mRNA (detected using probes targeting mouse *ALPL* on chromosome 4) is downregulated in JAK2^V617F^ MKs, the PMF overall condition might stabilize the protein isoform expressed in MKs, and/or MK Alpl protein half life is far greater than its mRNA. Although both WT and JAK2^V617F^ MK -derived CM had a tendency to inhibit OB differentiation, no significant difference was observed between them. We speculate that MKs act by both secreted factors and *via* direct cell contact. Nevertheless, the direct cell contact may enhance the inhibitory effect of JAK2^V617F^ MKs. It was interesting that Noggin, which is a secreted protein, was highly elevated in the JAK2^V617F^ MKs, yet there was no difference regarding the effect of WT- and JAK2^V617F^-MK derived CM on OB differentiation. In our OB/MK-CM system, we kept the CM for three days in culture. A possible explanation is that Noggin protein has a short lifetime and its continuous production by JAK2^V617F^ MKs is necessary to exert the higher inhibitory effect.

For each gene or protein differentially displayed in JAK2^V617F^ mutated MKs compared to controls, we contemplated cause-and-effect studies to examine their contribution to the inhibitory effect of male MKs on OB differentiation. Yet, it became apparent that the mutated MKs undergo a change in global gene programing involving several pathways linked to inhibition of osteogenesis. Hence, we propose that a cluster of such changes, and not a single one, is promoting the inhibition observed in OB differentiation. The observation that femurs from male JAK2^V617F^ had significantly reduced bone volume compared to age-matched controls corroborates the *in vitro* data of an inhibitory effect of their MKs on OB differentiation. In addition, the hypomineralized bone in JAK2^V617F^ mice further supports the notion of a compromised OB differentiation, since not fully differentiated OBs cannot complete the mineralization process. Yet, we recognize that the influence of JAK2^V617F^ mutated MKs on OB differentiation, while significant, might be a partial reason for the observed bone phenotype, as other factors and physiological conditions associated with PMF might contribute to it.

While our study points to a significant inhibitory effect of the JAK2^V617F^ mutation on bone volume in male mice, it is important to note that the final outcome of bone homeostasis is a result of both OB and OC regulation. More studies are needed to explore possible effects of JAK2^V617F^ MKs on OC differentiation, if at all. Future studies could also focus on examining the effect of sex on bone manifestations in other forms of MPN.

## Data Availability Statement

The original contributions presented in the study are included in the article/**Supplementary Material**. Further inquiries can be directed to the corresponding author.

## Ethics Statement

The animal study was reviewed and approved by Boston University Medical Campus Institutional Animal Care and Use Committee.

## Author Contributions

KR and AK generated the hypotheses. AK designed and performed most of the experiments, analysed data and wrote the manuscript with KR’s guidance, input and editing throughout the study. SM advised on experiments, performed flow cytometry experiments and analysis, and provided general insights on manuscript writing. LG assisted with RGB trichrome staining of bone sections and provided insights. AK wrote the initial draft of the manuscript and all authors contributed to different degrees to editing and finalizing the texts and figures.

## Funding

KR and AK are supported by NIH/NHLBI grants R01 HL136363 and R01 HL158670. SM is supported by NIH/ORIP K01 OD025290 grant.

## Conflict of Interest

The authors declare that the research was conducted in the absence of any commercial or financial relationships that could be construed as a potential conflict of interest.

## Publisher’s Note

All claims expressed in this article are solely those of the authors and do not necessarily represent those of their affiliated organizations, or those of the publisher, the editors and the reviewers. Any product that may be evaluated in this article, or claim that may be made by its manufacturer, is not guaranteed or endorsed by the publisher.

## References

[1] Titmarsh GJ Duncombe AS McMullin MF O'Rorke M Mesa R De Vocht F. How Common are Myeloproliferative Neoplasms? A Systematic Review and Meta-Analysis. Am J Hematol (2014) 89(6):581–7. doi: 10.1002/ajh.23690 24971434

[2] Szuber N Mudireddy M Nicolosi M Penna D Vallapureddy RR Lasho TL. 3023 Mayo Clinic Patients With Myeloproliferative Neoplasms: Risk-Stratified Comparison of Survival and Outcomes Data Among Disease Subgroups. Mayo Clinic Proc (2019) 94(4):599–610. doi: 10.1016/j.mayocp.2018.08.022 30824279

[3] Tefferi A. Primary Myelofibrosis: 2021 Update on Diagnosis, Risk-Stratification and Management. Am J Hematol (2021) 96(1):145–62. doi: 10.1002/ajh.26050 33197049

[4] Karagianni A Ravid K. Myeloproliferative Disorders and Their Effects on Bone Homeostasis: The Role of Megakaryocytes. Blood (2022) 139(21):3127–37. doi: 10.1182/blood.2021011480 PMC9136883 34428274

[5] Huang W Yang S Shao J Li YP. Signaling and Transcriptional Regulation in Osteoblast Commitment and Differentiation. Front Biosci (2007) 12:3068–92. doi: 10.2741/2296 PMC3571113 17485283

[6] Fox SB Lorenzen J Heryet A Jones M Gatter KC Mason DY. Megakaryocytes in Myelodysplasia: An Immunohistochemical Study on Bone Marrow Trephines. Histopathology (1990) 17(1):69–74. doi: 10.1111/j.1365-2559.1990.tb00665.x 1699866

[7] Thiele J Quitmann H Wagner S Fischer R. Dysmegakaryopoiesis in Myelodysplastic Syndromes (MDS): An Immunomorphometric Study of Bone Marrow Trephine Biopsy Specimens. J Clin Pathol (1991) 44(4):300–5. doi: 10.1136/jcp.44.4.300 PMC496903 2030148

[8] Ghai S Rai S. Megakaryocytic Morphology in Janus Kinase 2 V617F Positive Myeloproliferative Neoplasm. South Asian J Cancer (2017) 6(2):75–8. doi: 10.4103/2278-330X.208854 PMC5506815 28702412

[9] Kacena MA Shivdasani RA Wilson K Xi Y Troiano N Nazarian A. Megakaryocyte-Osteoblast Interaction Revealed in Mice Deficient in Transcription Factors GATA-1 and NF-E2. J Bone Mineral Res (2004) 19(4):652–60. doi: 10.1359/JBMR.0301254 15005853

[10] Vannucchi AM Bianchi L Cellai C Paoletti F Rana RA Lorenzini R. Development of Myelofibrosis in Mice Genetically Impaired for GATA-1 Expression (GATA-1(Low) Mice). Blood (2002) 100(4):1123–32. doi: 10.1182/blood-2002-06-1913 12149188

[11] Kacena MA Gundberg CM Nelson T Horowitz MC. Loss of the Transcription Factor P45 NF-E2 Results in a Developmental Arrest of Megakaryocyte Differentiation and the Onset of a High Bone Mass Phenotype. Bone (2005) 36(2):215–23. doi: 10.1016/j.bone.2004.09.024 15780947

[12] Kakumitsu H Kamezaki K Shimoda K Karube K Haro T Numata A. Transgenic Mice Overexpressing Murine Thrombopoietin Develop Myelofibrosis and Osteosclerosis. Leuk Res (2005) 29(7):761–9. doi: 10.1016/j.leukres.2004.12.009 15927672

[13] Villeval JL Cohen-Solal K Tulliez M Giraudier S Guichard J Burstein SA. High Thrombopoietin Production by Hematopoietic Cells Induces a Fatal Myeloproliferative Syndrome in Mice. Blood (1997) 90(11):4369–83. doi: 10.1182/blood.V90.11.4369 9373248

[14] Meijome TE Hooker RA Cheng YH Walker W Horowitz MC Fuchs RK. GATA-1 Deficiency Rescues Trabecular But Not Cortical Bone in OPG Deficient Mice. J Cell Physiol (2015) 230(4):783–90. doi: 10.1002/jcp.24803 PMC4433552 25205203

[15] Kacena MA Gundberg CM Kacena WJ 3rd Landis WJ Boskey AL Bouxsein ML. The Effects of GATA-1 and NF-E2 Deficiency on Bone Biomechanical, Biochemical, and Mineral Properties. J Cell Physiol (2013) 228(7):1594–600. doi: 10.1002/jcp.24322 PMC4128339 23359245

[16] Alvarez MB Xu L Childress PJ Maupin KA Mohamad SF Chitteti BR. Megakaryocyte and Osteoblast Interactions Modulate Bone Mass and Hematopoiesis. Stem Cells Dev (2018) 27(10):671–82. doi: 10.1089/scd.2017.0178 PMC5962330 29631496

[17] Stavnichuk M Tauer JT Nagy Z Mazharian A Welman M Lordkipanidzé M. Severity of Megakaryocyte-Driven Osteosclerosis in Mpig6b-Deficient Mice Is Sex-Linked. J Bone Mineral Res (2021) 36(4):803–13. doi: 10.1002/jbmr.4245 33434328

[18] Suva LJ Hartman E Dilley JD Russell S Akel NS Skinner RA. Platelet Dysfunction and a High Bone Mass Phenotype in a Murine Model of Platelet-Type Von Willebrand Disease. Am J Pathol (2008) 172(2):430–9. doi: 10.2353/ajpath.2008.070417 PMC2312362 18187573

[19] Olivos DJ 3rd Alvarez M Cheng YH Hooker RA Ciovacco WA Bethel M. Lnk Deficiency Leads to TPO-Mediated Osteoclastogenesis and Increased Bone Mass Phenotype. J Cell Biochem (2017) 118(8):2231–40. doi: 10.1002/jcb.25874 PMC5562279 28067429

[20] Ciovacco WA Goldberg CG Taylor AF Lemieux JM Horowitz MC Donahue HJ. The Role of Gap Junctions in Megakaryocyte-Mediated Osteoblast Proliferation and Differentiation. Bone (2009) 44(1):80–6. doi: 10.1016/j.bone.2008.08.117 PMC2659565 18848655

[21] Emmakah AM Arman HE Alvarez MB Childress PJ Bidwell JP Goebel WS. Megakaryocytes Enhance Mesenchymal Stromal Cells Proliferation and Inhibit Differentiation. J Cell Biochem (2017). doi: 10.1002/jcb.26289 28722829

[22] Lee YS Kwak MK Moon SA Choi YJ Baek JE Park SY. Regulation of Bone Metabolism by Megakaryocytes in a Paracrine Manner. Sci Rep (2020) 10(1):2277. doi: 10.1038/s41598-020-59250-6 32042021 PMC7010738

[23] Cheng YH Hooker RA Nguyen K Gerard-O'Riley R Waning DL Chitteti BR. Pyk2 Regulates Megakaryocyte-Induced Increases in Osteoblast Number and Bone Formation. J Bone Mineral Res (2013) 28(6):1434–45. doi: 10.1002/jbmr.1876 PMC3663900 23362087

[24] Tang Y Hu M Xu Y Chen F Chen S Chen M. Megakaryocytes Promote Bone Formation Through Coupling Osteogenesis With Angiogenesis by Secreting TGF-β1. Theranostics (2020) 10(5):2229–42. doi: 10.7150/thno.40559 PMC7019172 32104505

[25] Bord S Frith E Ireland DC Scott MA Craig JI Compston JE. Megakaryocytes Modulate Osteoblast Synthesis of Type-L Collagen, Osteoprotegerin, and RANKL. Bone (2005) 36(5):812–9. doi: 10.1016/j.bone.2004.12.006 15794927

[26] Xing S Wanting TH Zhao W Ma J Wang S Xu X. Transgenic Expression of JAK2V617F Causes Myeloproliferative Disorders in Mice. Blood (2008) 111(10):5109–17. doi: 10.1182/blood-2007-05-091579 PMC2384138 18334677

[27] Gaytan F Morales C Reymundo C Tena-Sempere M. A Novel RGB-Trichrome Staining Method for Routine Histological Analysis of Musculoskeletal Tissues. Sci Rep (2020) 10(1):16659. doi: 10.1038/s41598-020-74031-x 33028938 PMC7541469

[28] Lucero HA Patterson S Matsuura S Ravid K. Quantitative Histological Image Analyses of Reticulin Fibers in a Myelofibrotic Mouse. J Biol Methods (2016) 3(4):e60. doi: 10.14440/jbm.2016.152 28008415 PMC5172452

[29] Drachman JG Sabath DF Fox NE Kaushansky K. Thrombopoietin Signal Transduction in Purified Murine Megakaryocytes. Blood (1997) 89(2):483–92. doi: 10.1182/blood.V89.2.483 9002950

[30] Carroll SH Wigner NA Kulkarni N Johnston-Cox H Gerstenfeld LC Ravid K. A2B Adenosine Receptor Promotes Mesenchymal Stem Cell Differentiation to Osteoblasts and Bone Formation In Vivo. J Biol Chem (2012) 287(19):15718–27. doi: 10.1074/jbc.M112.344994 PMC3346096 22403399

[31] Edgar CM Chakravarthy V Barnes G Kakar S Gerstenfeld LC Einhorn TA. Autogenous Regulation of a Network of Bone Morphogenetic Proteins (BMPs) Mediates the Osteogenic Differentiation in Murine Marrow Stromal Cells. Bone (2007) 40(5):1389–98. doi: 10.1016/j.bone.2007.01.001 PMC2681090 17303481

[32] Leiva O Ng SK Matsuura S Chitalia V Lucero H Findlay A. Novel Lysyl Oxidase Inhibitors Attenuate Hallmarks of Primary Myelofibrosis in Mice. Int J Hematol (2019) 110(6):699–708. doi: 10.1007/s12185-019-02751-6 31637674 PMC7503219

[33] Matsuura S Thompson CR Belghasem ME Bekendam RH Piasecki A Leiva O. Platelet Dysfunction and Thrombosis in JAK2(V617F)-Mutated Primary Myelofibrotic Mice. Arterioscler Thromb Vasc Biol (2020) 40(10):e262–e72. doi: 10.1161/ATVBAHA.120.314760 PMC7605151 32814440

[34] Ciovacco WA Cheng YH Horowitz MC Kacena MA. Immature and Mature Megakaryocytes Enhance Osteoblast Proliferation and Inhibit Osteoclast Formation. J Cell Biochem (2010) 109(4):774–81. doi: 10.1002/jcb.22456 PMC3095430 20052670

[35] Stavnichuk M Komarova SV. Megakaryocyte-Bone Cell Interactions: Lessons From Mouse Models of Experimental Myelofibrosis and Related Disorders. Am J Physiol Cell Physiol (2022) 322(2):C177–c84. doi: 10.1152/ajpcell.00328.2021 34910601

[36] Bourebaba L Marycz K. Pathophysiological Implication of Fetuin-A Glycoprotein in the Development of Metabolic Disorders: A Concise Review. J Clin Med (2019) 8(12):2033. doi: 10.3390/jcm8122033 PMC6947209 31766373

[37] Schinke T Amendt C Trindl A Pöschke O Müller-Esterl W Jahnen-Dechent W. The Serum Protein Alpha2-HS Glycoprotein/Fetuin Inhibits Apatite Formation *In Vitro* and in Mineralizing Calvaria Cells. A Possible Role in Mineralization and Calcium Homeostasis. J Biol Chem (1996) 271(34):20789–96. doi: 10.1074/jbc.271.34.20789 8702833

[38] Demetriou M Binkert C Sukhu B Tenenbaum HC Dennis JW. Fetuin/alpha2-HS Glycoprotein is a Transforming Growth Factor-Beta Type II Receptor Mimic and Cytokine Antagonist. J Biol Chem (1996) 271(22):12755–61. doi: 10.1074/jbc.271.22.12755 8662721

[39] Szweras M Liu D Partridge EA Pawling J Sukhu B Clokie C. Alpha 2-HS Glycoprotein/Fetuin, a Transforming Growth Factor-Beta/Bone Morphogenetic Protein Antagonist, Regulates Postnatal Bone Growth and Remodeling. J Biol Chem (2002) 277(22):19991–7. doi: 10.1074/jbc.M112234200 11901155

[40] Vimalraj S. Alkaline Phosphatase: Structure, Expression and its Function in Bone Mineralization. Gene (2020) 754:144855. doi: 10.1016/j.gene.2020.144855 32522695

[41] Fedde KN Blair L Silverstein J Coburn SP Ryan LM Weinstein RS. Alkaline Phosphatase Knock-Out Mice Recapitulate the Metabolic and Skeletal Defects of Infantile Hypophosphatasia. J Bone Mineral Res (1999) 14(12):2015–26. doi: 10.1359/jbmr.1999.14.12.2015 PMC3049802 10620060

[42] Petryk A Shimmi O Jia X Carlson AE Tervonen L Jarcho MP. Twisted Gastrulation and Chordin Inhibit Differentiation and Mineralization in MC3T3-E1 Osteoblast-Like Cells. Bone (2005) 36(4):617–26. doi: 10.1016/j.bone.2005.01.018 15780974

[43] Wang C Xiao F Gan Y Yuan W Zhai Z Jin T. Improving Bone Regeneration Using Chordin siRNA Delivered by pH-Responsive and Non-Toxic Polyspermine Imidazole-4,5-Imine. Cell Physiol Biochem (2018) 46(1):133–47. doi: 10.1159/000488416 29587276

[44] Volk SW Shah SR Cohen AJ Wang Y Brisson BK Vogel LK. Type III Collagen Regulates Osteoblastogenesis and the Quantity of Trabecular Bone. Calcif Tissue Int (2014) 94(6):621–31. doi: 10.1007/s00223-014-9843-x PMC4335719 24626604

[45] Yen JL Lin SP Chen MR Niu DM. Clinical Features of Ehlers-Danlos Syndrome. J Formosan Med Assoc (2006) 105(6):475–80. doi: 10.1016/S0929-6646(09)60187-X 16801035

[46] Hopwood B Tsykin A Findlay DM Fazzalari NL. Gene Expression Profile of the Bone Microenvironment in Human Fragility Fracture Bone. Bone (2009) 44(1):87–101. doi: 10.1016/j.bone.2008.08.120 18840552

[47] Hong D Chen H-X Yu H-Q Liang Y Wang C Lian Q-Q. Morphological and Proteomic Analysis of Early Stage of Osteoblast Differentiation in Osteoblastic Progenitor Cells. Exp Cell Res (2010) 316(14):2291–300. doi: 10.1016/j.yexcr.2010.05.011 PMC4580249 20483354

[48] Paralkar VM Nandedkar AK Pointer RH Kleinman HK Reddi AH. Interaction of Osteogenin, a Heparin Binding Bone Morphogenetic Protein, With Type IV Collagen. J Biol Chem (1990) 265(28):17281–4. doi: 10.1016/S0021-9258(17)44900-3 2211625

[49] Paralkar VM Vukicevic S Reddi AH. Transforming Growth Factor Beta Type 1 Binds to Collagen IV of Basement Membrane Matrix: Implications for Development. Dev Biol (1991) 143(2):303–8. doi: 10.1016/0012-1606(91)90081-D 1991553

[50] Heo JS Lee SG Kim HO. Distal-Less Homeobox 5 is a Master Regulator of the Osteogenesis of Human Mesenchymal Stem Cells. Int J Mol Med (2017) 40(5):1486–94. doi: 10.3892/ijmm.2017.3142 PMC5627883 28949384

[51] Robledo RF Rajan L Li X Lufkin T. The Dlx5 and Dlx6 Homeobox Genes are Essential for Craniofacial, Axial, and Appendicular Skeletal Development. Genes Dev (2002) 16(9):1089–101. doi: 10.1101/gad.988402 PMC186247 12000792

[52] Wu XB Li Y Schneider A Yu W Rajendren G Iqbal J. Impaired Osteoblastic Differentiation, Reduced Bone Formation, and Severe Osteoporosis in Noggin-Overexpressing Mice. J Clin Invest (2003) 112(6):924–34. doi: 10.1172/JCI15543 PMC193662 12975477

[53] Devlin RD Du Z Pereira RC Kimble RB Economides AN Jorgetti V. Skeletal Overexpression of Noggin Results in Osteopenia and Reduced Bone Formation. Endocrinology (2003) 144(5):1972–8. doi: 10.1210/en.2002-220918 12697704

[54] Walsh DW Godson C Brazil DP Martin F. Extracellular BMP-Antagonist Regulation in Development and Disease: Tied Up in Knots. Trends Cell Biol (2010) 20(5):244–56. doi: 10.1016/j.tcb.2010.01.008 20188563

[55] Chang AC Chen PC Lin YF Su CM Liu JF Lin TH. Osteoblast-Secreted WISP-1 Promotes Adherence of Prostate Cancer Cells to Bone *via* the VCAM-1/Integrin α4β1 System. Cancer Lett (2018) 426:47–56. doi: 10.1016/j.canlet.2018.03.050 29627497

[56] Marie PJ. Targeting Integrins to Promote Bone Formation and Repair. Nat Rev Endocrinol (2013) 9(5):288–95. doi: 10.1038/nrendo.2013.4 23358353

[57] Sens C Huck K Pettera S Uebel S Wabnitz G Moser M. Fibronectins Containing Extradomain A or B Enhance Osteoblast Differentiation *via* Distinct Integrins. J Biol Chem (2017) 292(19):7745–60. doi: 10.1074/jbc.M116.739987 PMC5427257 28325836

[58] Krause C Guzman A Knaus P. Noggin. Int J Biochem Cell Biol (2011) 43(4):478–81. doi: 10.1016/j.biocel.2011.01.007 21256973

[59] Beederman M Lamplot JD Nan G Wang J Liu X Yin L. BMP Signaling in Mesenchymal Stem Cell Differentiation and Bone Formation. J BioMed Sci Eng (2013) 6(8A):32–52. doi: 10.4236/jbise.2013.68A1004 26819651 PMC4725591

[60] Oikonomidou PR Casu C Yang Z Crielaard B Shim JH Rivella S. Polycythemia is Associated With Bone Loss and Reduced Osteoblast Activity in Mice. Osteoporosis Int (2016) 27(4):1559–68. doi: 10.1007/s00198-015-3412-7 PMC5319412 26650379

[61] Mullally A Lane SW Ball B Megerdichian C Okabe R Al-Shahrour F. Physiological Jak2V617F Expression Causes a Lethal Myeloproliferative Neoplasm With Differential Effects on Hematopoietic Stem and Progenitor Cells. Cancer Cell (2010) 17(6):584–96. doi: 10.1016/j.ccr.2010.05.015 PMC2909585 20541703

[62] Adam S Simon N Steffen U Andes FT Scholtysek C Müller DIH. JAK Inhibition Increases Bone Mass in Steady-State Conditions and Ameliorates Pathological Bone Loss by Stimulating Osteoblast Function. Sci Trans Med (2020) 12(530):eaay4447. doi: 10.1126/scitranslmed.aay4447 32051226

[63] Bais MV Wigner N Young M Toholka R Graves DT Morgan EF. BMP2 is Essential for Post Natal Osteogenesis But Not for Recruitment of Osteogenic Stem Cells. Bone (2009) 45(2):254–66. doi: 10.1016/j.bone.2009.04.239 PMC2745982 19398045

[64] Gerard PB. Megakaryocytes: Morphogenesis, Biochemistry and Physiology, a Review. Bios (1974) 45(2):59–68.

[65] Kerppola W. Observations on the Phosphatase Content of Blood and Bone Marrow Cells in Normal and Pathologic Hemopoiesis. Blood (1951) 6(5):454–65. doi: 10.1182/blood.V6.5.454.454 14830640

[66] Erber WN Breton-Gorius J Villeval JL Oscier DG Bai Y Mason DY. Detection of Cells of Megakaryocyte Lineage in Haematological Malignancies by Immuno-Alkaline Phosphatase Labelling Cell Smears With a Panel of Monoclonal Antibodies. Br J Haematol (1987) 65(1):87–94. doi: 10.1111/j.1365-2141.1987.tb06140.x 3545280

[67] Erber WN. Immunocytochemical Labelling of Haematological Samples Using Monoclonal Antibodies. Cells (2021) 11(1):127. doi: 10.3390/cells11010127 35011689 PMC8750895

