# Supplementary material for: Inhibition of Osteoblast Differentiation by JAK2V617F Megakaryocytes Derived From Male Mice With Primary Myelofibrosis
Source: Front Oncol. 2022 Jul 8;12:929498. doi: 10.3389/fonc.2022.929498 (PMC9307716; doi:10.3389/fonc.2022.929498)
Supplement: Supplementary file 1 [file DataSheet_1.pdf]

Supplementary Figure 1

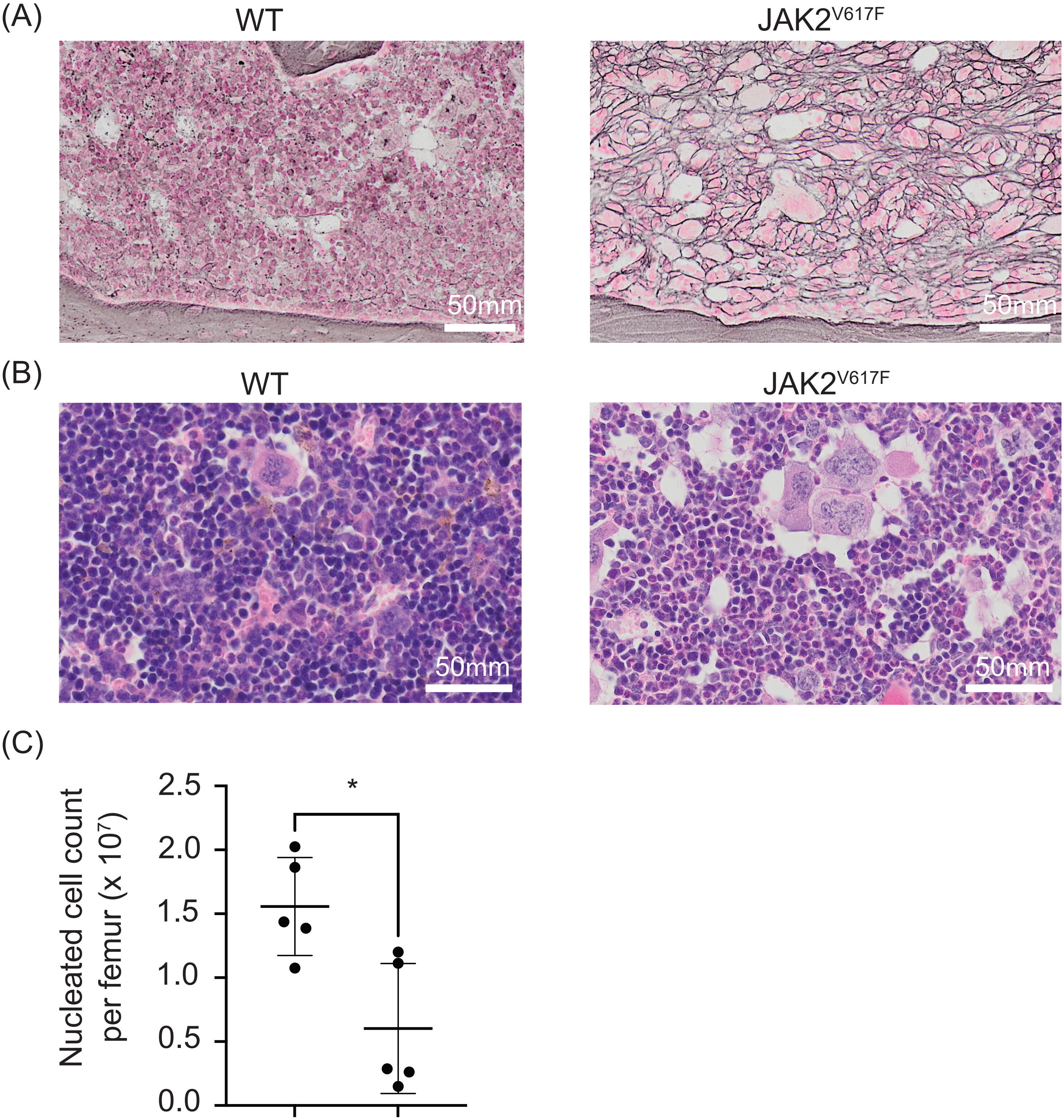

Supplementary Figure 1: JAK2^{V617F} mice show myelofibrosis and hypocellular bone marrow. **(A)** Representative images of bone sections of WT and JAK2^{V617F} mice subjected to modified Gomori's silver staining to detect reticulin fibers, appearing as black strings. **(B)** Representative images of bone sections of WT and JAK2^{V617F} mice stained for hematoxylin & eosin. **(C)** Bone marrow nucleated cell count. Five JAK2^{V617F} and five WT 30 weeks old male mice were analyzed. *Denotes $p<0.05$
