# Supplementary material for: Inhibition of Osteoblast Differentiation by JAK2V617F Megakaryocytes Derived From Male Mice With Primary Myelofibrosis
Source: Front Oncol. 2022 Jul 8;12:929498. doi: 10.3389/fonc.2022.929498 (PMC9307716; doi:10.3389/fonc.2022.929498)
Supplement: Supplementary file 2 [file DataSheet_2.pdf]

Supplementary Figure 2

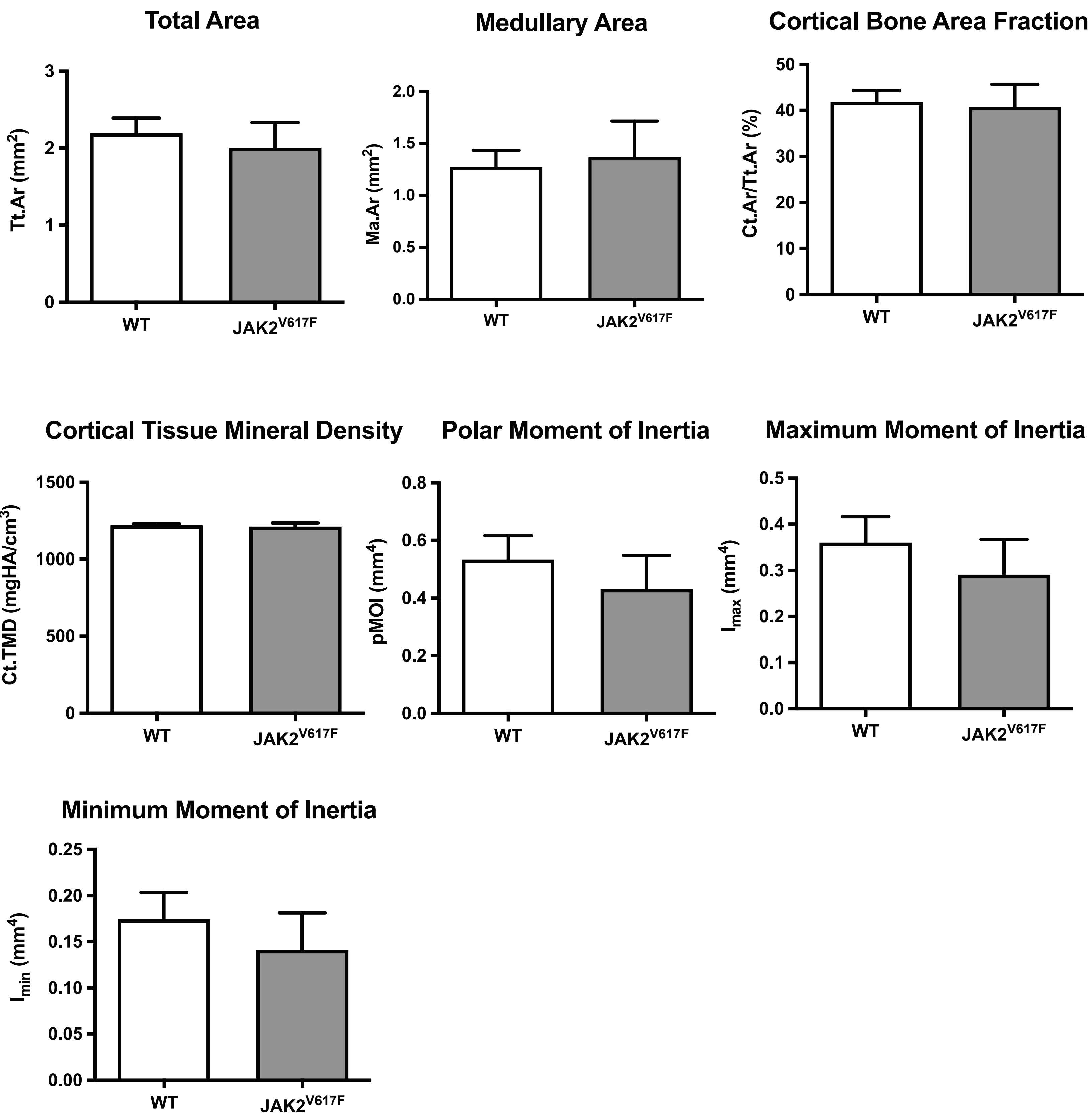

Supplementary Figure 2: Quantitative micro-CT analysis of cortical structure (continuation of Figure 1C). Measurements include total cross-sectional area (bone + medullary area) (Tt.Ar, mm^2), medullary area (Ma.Ar, mm^2), bone area fraction (Ct.Ar/Tt.Ar, %), cortical tissue mineral density (Ct.TMD, $mgHA/cm^3$), as well as maximum, minimum and polar moments of inertia (Imax, Imin, and J, mm^4). Measurements are presented as mean±SD. Seven $JAK2^{V617F}$ and six control 30 weeks old mice were analyzed. An unpaired two-tailed t-test comparing the $JAK2^{V617F}$ and WT groups gave no statistically significant p-values.
