# Supplementary material for: Inhibition of Osteoblast Differentiation by JAK2V617F Megakaryocytes Derived From Male Mice With Primary Myelofibrosis
Source: Front Oncol. 2022 Jul 8;12:929498. doi: 10.3389/fonc.2022.929498 (PMC9307716; doi:10.3389/fonc.2022.929498)
Supplement: Supplementary file 3 [file DataSheet_3.pdf]

Supplementary Figure 3

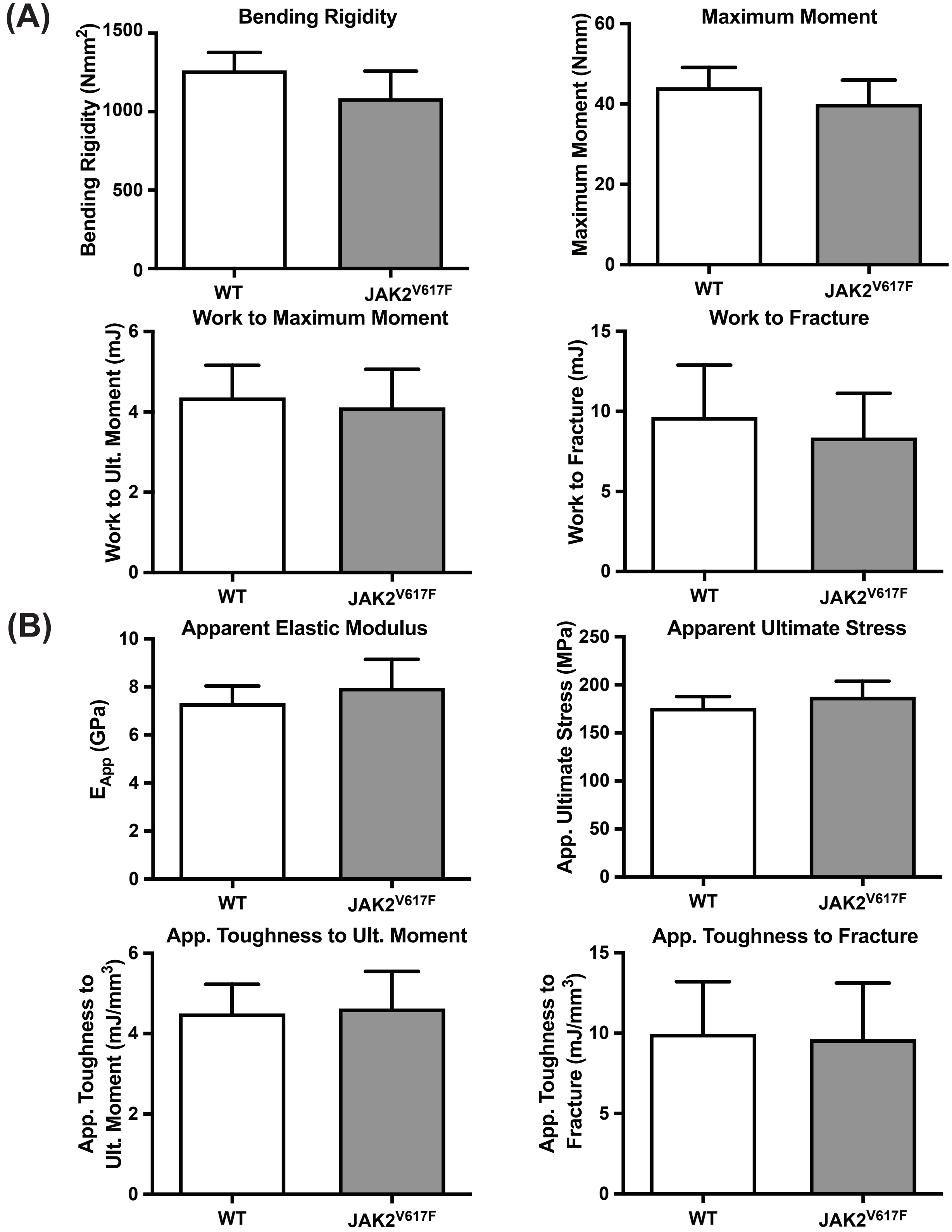

Supplementary Figure 3: Three-point bending of the femoral diaphysis from the same femurs used for micro-CT and histology. This assay determines the effect of constitutively active JAK2 expression on bone mechanical properties. **(A)** Whole bone mechanical properties: bending rigidity (Nmm^2), maximum moment (Nmm), work to maximum moment (mJ) and work to fracture (mJ) **(B)** Tissue level mechanical properties (Apparent material properties): apparent elastic modulus (GPa), apparent ultimate stress (MPa), apparent toughness to ultimate moment (mJ/mm^3) and apparent toughness to fracture (mJ/mm^3). Plots are mean±SD. Seven JAK2^{V617F} and six control 30 weeks old mice were analyzed. An unpaired two-tailed t-test comparing the JAK2^{V617F} and WT groups gave no statistically significant p-values. It is possible that bone mechanical properties might be different in the two experimental groups upon inducing stress, such as fracture.
