# Supplementary material for: Inhibition of Osteoblast Differentiation by JAK2V617F Megakaryocytes Derived From Male Mice With Primary Myelofibrosis
Source: Front Oncol. 2022 Jul 8;12:929498. doi: 10.3389/fonc.2022.929498 (PMC9307716; doi:10.3389/fonc.2022.929498)
Supplement: Supplementary file 4 [file Table_1.docx]

**Supplementary Table 1: Complete blood count of mice used for micro-CT, three-point bending and histology (see Methods).** WBC, white blood cells; RBC, red blood cells; Hb, hemoglobin; HCT, hematocrit; PLT, platelets

| **Components** | **WBC (K/ul)** | **RBC (M/ul)** | | **Hb (g/dL)** | | **HCT (%)** | | **PLT (K/ul)** |
| --- | --- | --- | --- | --- | --- | --- | --- | --- |
| **WT**  (n=6) | 4.4±1.39 | 6.17±0.59 | 6.98±0.86 | | 32.87±2.77 | | 718.33±136.75 | |
| **JAK2^V617F^**  (n=7) | 11.68±5.16 | 6.87±1.38 | 6.71±1.76 | | 31.93±7.07 | | 1502.57±468.35 | |
| **p-value** | **0.01** | 0.25 | 0.73 | | 0.75 | | **0** | |
