# Supplementary material for: Inhibition of Osteoblast Differentiation by JAK2V617F Megakaryocytes Derived From Male Mice With Primary Myelofibrosis
Source: Front Oncol. 2022 Jul 8;12:929498. doi: 10.3389/fonc.2022.929498 (PMC9307716; doi:10.3389/fonc.2022.929498)
Supplement: Supplementary file 5 [file Table_2.docx]

| **Supplementary Table 2: Ratio of spleen/body weight of mice used for micro-CT, three-point bending and histology (see Methods).** | | | | |
| --- | --- | --- | --- | --- |
| **Parameters** | **Body weight (g)** | **Weight of spleen (g)** | **Ratio**  **spleen/body weight** |  |
| **WT**  (n=6) | 33.03±2.56 | 0.08±0.006 | 0.002±0 |  |
| **JAK2^V617F^**  (n=7) | 29.07±2.35 | 0.88±0.08 | 0.03±0.002 |  |
| **p-value** | **0.016** | **0.00** | **0.00** |  |
