# Supplementary material for: Inhibition of Osteoblast Differentiation by JAK2V617F Megakaryocytes Derived From Male Mice With Primary Myelofibrosis
Source: Front Oncol. 2022 Jul 8;12:929498. doi: 10.3389/fonc.2022.929498 (PMC9307716; doi:10.3389/fonc.2022.929498)
Supplement: Supplementary file 6 [file Table_3.docx]

**Supplementary Table 3:** **Gene array analysis**. The whole gene panel of Commercial RT² Profiler™ PCR Array Mouse Osteogenesis PAMM-026Z. Fold up (“+”)- or down (“-”)-regulation of bone-development related mRNA level in JAK2^V617F^ MKs vs. WT MKs. Four JAK2^V617F^ and four WT male mice, 17 weeks of age were analyzed. MKs derived from 2 mice were combined as one sample to obtain sufficient material and increase biological representation, and, thus, 2 samples were tested per category.

| **Gene** | | **Fold-up (+) or down (-) regulation of mRNA**  **in JAK2^V617F^/WT** |
| --- | --- | --- |
| **Acvr1** | Activin A receptor, type 1 | -1.28 |
| **Ahsg** | Alpha-2-HS-glycoprotein | +8.66 |
| **Alpl** | Alkaline phosphatase, liver/bone/kidney | -22.81 |
| **Anxa5** | Annexin A5 | -1.14 |
| **Bglap** | Bone gamma carboxyglutamate protein | +1.23 |
| **Bgn** | Biglycan | -1.54 |
| **Bmp1** | Bone morphogenetic protein 1 | -1.52 |
| **Bmp2** | Bone morphogenetic protein 2 | +3.52 |
| **Bmp3** | Bone morphogenetic protein 3 | -2.34 |
| **Bmp4** | Bone morphogenetic protein 4 | +2.89 |
| **Bmp5** | Bone morphogenetic protein 5 | -1.66 |
| **Bmp6** | Bone morphogenetic protein 6 | +1.28 |
| **Bmp7** | Bone morphogenetic protein 7 | -1.38 |
| **Bmpr1a** | Bone morphogenetic protein receptor, type 1A | -1.05 |
| **Bmpr1b** | Bone morphogenetic protein receptor, type 1B | +1.23 |
| **Bmpr2** | Bone morphogenic protein receptor, type II (serine/threonine kinase) | +2.23 |
| **Cd36** | CD36 antigen | -3.73 |
| **Cdh11** | Cadherin 11 | -1.29 |
| **Chrd** | Chordin | +10.47 |
| **Col10a1** | Collagen, type X, alpha 1 | +1.68 |
| **Col14a1** | Collagen, type XIV, alpha 1 | +6.50 |
| **Col1a1** | Collagen, type I, alpha 1 | -1.95 |
| **Col1a2** | Collagen, type I, alpha 2 | -1.77 |
| **Col2a1** | Collagen, type II, alpha 1 | -3.53 |
| **Col3a1** | Collagen, type III, alpha 1 | -4.06 |
| **Col4a1** | Collagen, type IV, alpha 1 | +6.58 |
| **Col5a1** | Collagen, type V, alpha 1 | -1.82 |
| **Comp** | Cartilage oligomeric matrix protein | +3.14 |
| **Csf1** | Colony stimulating factor 1 (macrophage) | -1.63 |
| **Csf2** | Colony stimulating factor 2 (granulocyte-macrophage) | +1.32 |
| **Csf3** | Colony stimulating factor 3 (granulocyte) | -3.11 |
| **Ctsk** | Cathepsin K | -1.23 |
| **Dlx5** | Distal-less homeobox 5 | -4.25 |
| **Egf** | Epidermal growth factor | +1.21 |
| **Fgf1** | Fibroblast growth factor 1 | +1.16 |
| **Fgf2** | Fibroblast growth factor 2 | -3.39 |
| **Fgfr1** | Fibroblast growth factor receptor 1 | +1.71 |
| **Fgfr2** | Fibroblast growth factor receptor 2 | -3.47 |
| **Flt1** | FMS-like tyrosine kinase 1 | +1.71 |
| **Fn1** | Fibronectin 1 | +1.92 |
| **Gdf10** | Growth differentiation factor 10 | -1.02 |
| **Gli1** | GLI-Kruppel family member GLI1 | +2.38 |
| **Icam1** | Intercellular adhesion molecule 1 | -3.89 |
| **Igf1** | Insulin-like growth factor 1 | -3.83 |
| **Igf1r** | Insulin-like growth factor I receptor | +1.07 |
| **Ihh** | Indian hedgehog | +1.38 |
| **Itga2** | Integrin alpha 2 | -1.76 |
| **Itga2b** | Integrin alpha 2b | -1.09 |
| **Itga3** | Integrin alpha 3 | -1.78 |
| **Itgam** | Integrin alpha M | -1.96 |
| **Itgav** | Integrin alpha V | -1.17 |
| **Itgb1** | Integrin beta 1 (fibronectin receptor beta) | -1.10 |
| **Mmp10** | Matrix metallopeptidase 10 | -3.84 |
| **Mmp2** | Matrix metallopeptidase 2 | +1.15 |
| **Mmp8** | Matrix metallopeptidase 8 | +1.08 |
| **Mmp9** | Matrix metallopeptidase 9 | -1.32 |
| **Nfkb1** | Nuclear factor of kappa light polypeptide gene enhancer in B-cells 1, p105 | +2.29 |
| **Nog** | Noggin | +19.85 |
| **Pdgfa** | Platelet derived growth factor, alpha | -1.20 |
| **Phex** | Phosphate regulating gene with homologies to endopeptidases on the X chromosome (hypophosphatemia, vitamin D resistant rickets) | +1.15 |
| **Runx2** | Runt related transcription factor 2 | -1.17 |
| **Serpinh1** | Serine (or cysteine) peptidase inhibitor, clade H, member 1 | +1.19 |
| **Smad1** | MAD homolog 1 (Drosophila) | -1.35 |
| **Smad2** | MAD homolog 2 (Drosophila) | +1.26 |
| **Smad3** | MAD homolog 3 (Drosophila) | +1.06 |
| **Smad4** | MAD homolog 4 (Drosophila) | +1.27 |
| **Smad5** | MAD homolog 5 (Drosophila) | +1.03 |
| **Sost** | Sclerostin | -4.30 |
| **Sox9** | SRY-box containing gene 9 | -1.73 |
| **Sp7** | Sp7 transcription factor 7 | -2.51 |
| **Spp1** | Secreted phosphoprotein 1 | -2.07 |
| **Tgfb1** | Transforming growth factor, beta 1 | -1.29 |
| **Tgfb2** | Transforming growth factor, beta 2 | -1.61 |
| **Tgfb3** | Transforming growth factor, beta 3 | -1.07 |
| **Tgfbr1** | Transforming growth factor, beta receptor I | -1.20 |
| **Tgfbr2** | Transforming growth factor, beta receptor II | -1.53 |
| **Tgfbr3** | Transforming growth factor, beta receptor III | -1.27 |
| **Tnf** | Tumor necrosis factor | -2.73 |
| **Tnfsf11** | Tumor necrosis factor (ligand) superfamily, member 11 | -2.38 |
| **Twist1** | Twist homolog 1 (Drosophila) | +1.14 |
| **Vcam1** | Vascular cell adhesion molecule 1 | -7.45 |
| **Vdr** | Vitamin D receptor | -2.11 |
| **Vegfa** | Vascular endothelial growth factor A | +2.71 |
| **Vegfb** | Vascular endothelial growth factor B | -1.44 |
